# CBX7 Modulates the Expression of Genes Critical for Cancer Progression

**DOI:** 10.1371/journal.pone.0098295

**Published:** 2014-05-27

**Authors:** Pierlorenzo Pallante, Romina Sepe, Antonella Federico, Floriana Forzati, Mimma Bianco, Alfredo Fusco

**Affiliations:** Istituto per l'Endocrinologia e l'Oncologia Sperimentale (IEOS), Consiglio Nazionale delle Ricerche (CNR), c/o Dipartimento di Medicina Molecolare e Biotecnologie Mediche (DMMBM), Università degli Studi di Napoli “Federico II”, Naples, Italy; Cincinnati Children's Hospital Medical Center, United States of America

## Abstract

**Background:**

We have previously shown that the expression of CBX7 is drastically decreased in several human carcinomas and that its expression progressively decreases with the appearance of a highly malignant phenotype. The aim of our study has been to investigate the mechanism by which the loss of CBX7 expression may contribute to the emergence of a more malignant phenotype.

**Methods:**

We analyzed the gene expression profile of a thyroid carcinoma cell line after the restoration of CBX7 and, then, analyzed the transcriptional regulation of identified genes. Finally, we evaluated the expression of CBX7 and regulated genes in a panel of thyroid and lung carcinomas.

**Results:**

We found that CBX7 negatively or positively regulates the expression of several genes (such as SPP1, SPINK1, STEAP1, and FOS, FOSB, EGR1, respectively) associated to cancer progression, by interacting with their promoter regions and modulating their transcriptional activity. Quantitative RT-PCR analyses in human thyroid and lung carcinoma tissues revealed a negative correlation between CBX7 and its down-regulated genes, while a positive correlation was observed with up-regulated genes.

**Conclusion:**

In conclusion, the loss of CBX7 expression might play a critical role in advanced stages of carcinogenesis by deregulating the expression of specific effector genes.

## Introduction

CBX7 is a Polycomb protein member of the polycomb repressive complex 1 (PRC1), a multiprotein complex that together with the polycomb repressive complex 2 (PRC2), maintains important developmental genes in a transcriptionally repressed state [1]–[3]. We have previously demonstrated that the CBX7 gene is drastically down-regulated in thyroid carcinomas and its expression progressively decreases with malignant grade and neoplastic stage [4]. Moreover, further studies have shown that the correlation of the loss of CBX7 with a highly malignant phenotype and a consequent poor prognosis is a general event in oncology. In fact, the loss of CBX7 expression has been recently shown to be associated with increasing malignancy grade in colon [5], bladder [6], pancreatic [7], breast [8], gastric [9] and lung carcinoma [10], whereas the retention of CBX7 expression correlates with a longer survival of the colon and pancreatic cancer patients [5], [7]. The restoration of CBX7 expression in thyroid, gastric and colon carcinoma cell lines inhibits cell growth with an accumulation of the cell population in the G1 phase of the cell cycle, suggesting a negative role of CBX7 in the control of the G1/S transition of the cell cycle.

It has been recently demonstrated that CBX7 is able to positively regulate the expression of the gene encoding the E-cadherin [11] that is known to play a critical role in maintaining normal epithelial cell morphology, and whose loss of expression represents a general feature of the epithelial-mesenchymal transition [12], [13]. In fact, it has been shown that CBX7 is able to preserve the expression of E-cadherin by interacting with histone deacetylase 2 and inhibiting its activity on the CDH1 promoter [11]. Consistently, a direct correlation between the levels of E-cadherin and CBX7 expression has been reported in thyroid and pancreatic carcinomas [7], [11]. Therefore, these results would suggest that the loss of the CBX7 gene expression may play a critical role in the late stages of human carcinoma progression.

The tumour suppressor role of CBX7 has been very recently confirmed by the phenotype of cbx7 knockout mice. Indeed, these mice develop liver and lung adenomas and carcinomas, and accordingly, mouse embryonic fibroblasts (MEFs) derived from the cbx7 knockout mice have a higher growth rate and a reduced susceptibility to senescence than their wild type counterparts [10]. Cyclin E overexpression, due to the lack of cbx7 that negatively regulates its expression, likely accounts for the phenotype of the cbx7 knockout mice. A similar mechanism is likely involved in human lung carcinogenesis since Cyclin E up-regulation, associated with the loss of CBX7 expression, has been observed in most of the human lung carcinomas analyzed [10].

The aim of the present study has been to investigate other mechanisms by which the loss of CBX7 expression may contribute to the appearance of a highly malignant phenotype. We first generated six thyroid carcinoma cell clones in which the expression of CBX7 has been restored, then, by using the powerful oligonucleotide microarray hybridization technique, we analysed the gene expression profile of the FRO cell line in which the expression of CBX7 was restored, compared to the same cell line not expressing CBX7.

We found that CBX7 was able to positively regulate 120 genes and negatively regulate 196 genes. Among them, we identified several specific effector genes, such as SPP1 (encoding the osteopontin protein), whose function is known to be essential for the acquisition of a fully malignant phenotype. Chromatin immunoprecipitation experiments demonstrated that CBX7 protein directly binds to the promoters of these regulated genes and functional studies confirmed that CBX7 expression was able to modulate the promoters of the CBX7-regulated genes. Finally, quantitative RT-PCR analyses showed an inverse correlation between CBX7 and its down-regulated genes and a positive correlation with its up-regulated ones in human thyroid and lung carcinoma samples at different degree of malignancy.

Taken together, the data reported here indicate that CBX7 negatively or positively controls the expression of several genes coding for proteins having a critical role in human cancer progression.

## Materials and Methods

### Microarray analysis

GeneChip Human Gene 1.0 ST Arrays (Affymetrix, Santa Clara, CA), consisting of 764,885 probe sets covering over 28,869 genes, was used to evaluate genes differentially expressed. The whole hybridization procedure was performed following the Affymetrix instructions. The amplification and labeling processes were verified using a GeneChip Eukaryotic Poly-A RNA Control Kit (Affymetrix) with exogenous positive controls. 15 µg of each biotinylated cRNA preparation was fragmented and placed in hybridization mixture containing biotinylated hybridization controls (GeneChip Expression Hybridization Controls, Affymetrix). Samples were then hybridized onto a GeneChip Human Gene 1.0 ST Array at 45 °C for 16 hours at constant rotation (60 rpm) in a Hybridization Oven (Affymetrix). Microarray scanned images were obtained with a GeneChip Scanner (Affymetrix) using the default settings. Images were analyzed with Affymetrix Gene Expression Analysis Software (Affymetrix). Comparisons were made between FRO-EV-1 and FRO-CBX7-1 samples, considering FRO-EV-1 as baseline. The fold change values, indicating the relative change in the expression levels between FRO-EV-1 and FRO-CBX7-1, were used to identify genes differentially expressed.

Microarray data are available in the ArrayExpress database (www.ebi.ac.uk/arrayexpress) under accession number E-MTAB-2420.

### Human tissue samples

The human thyroid cancer tissues, adjacent normal tissues or normal contralateral lobes, obtained from surgical specimens, were collected at the Service d'Anatomo-Pathologie, Centre Hospitalier Lyon Sud, Pierre Bénite, France, according to the guidelines of the ethics committee of this institute, and were immediately frozen in liquid nitrogen to subsequently perform the extraction of RNA.

“TissueScan Real-Time Lung Cancer Disease Panel III” of cDNAs was purchased from Origene (Origene Technologies Inc., Rockville, MD). This lung cancer cDNA panel (HLRT103) contains 8 normal lung specimens and 40 lung cancer specimens of different histotype, whose clinical pathological features are freely available at the following address: http://www.origene.com/qPCR/Tissue-qPCR-Arrays.aspx.

### RNA extraction, quantitative Real Time PCR (qRT-PCR) and Microarray analysis

Total RNA was extracted from cell lines and tissues using the RNAeasy Mini Kit (Qiagen, Valencia, CA). The integrity of the RNA was assessed by denaturing agarose gel electrophoresis. To generate cDNA, QuantiTect Reverse Transcription Kit (Qiagen) was used to reverse-transcribe 1 µg of total RNA from each sample. An optimized blend of oligo-dT and random primers was used.

Real-Time Quantitative PCR was carried out with the CFX 96 thermocycler (Bio-Rad, Hercules, CA) in 96-well plates using a final volume of 20 µl. For PCR we used 10 µl of 2× Sybr Green (Applied Biosystems, Foster City, CA), 200 nM of each primer, and cDNA generated from 20 ng of total RNA. The primers used are reported in Dataset S1. Thermal protocol was as follows: 2 min 95°C; then 45 cycles 20 s 95°C and 1 min 60°C. Each reaction was carried out in duplicate and the 2^−ΔΔCT^ method was employed to calculate relative expression levels [14]. G6PD was used as reference gene.

### Cell cultures and transfections

Thyroid carcinoma cells FRO [15] available in our laboratory and HEK 293 cells (American Type Culture Collection, LGC Standards S.r.l., Sesto San Giovanni, Italy) were grown in DMEM (Life Technologies, Grand Island, NY) supplemented with 10% fetal calf serum (Life Technologies), 1% glutamine, 1% penicillin/streptomycin (Life Technologies) in a 5% CO_2_ atmosphere. FRO and HEK 293 cells were transfected using either Lipofectamine reagent (Invitrogen, Carlsbad, CA) and Neon Electroporation System (Invitrogen) according to manufacturer's instructions. To generate CBX7 stable expressing cells, transfected cells were selected in a medium containing geneticin (Life Technologies), several clones were picked and expanded for further analyses. Rat normal thyroid PC Cl3 cells [16] were cultured in modified F12 medium supplemented with 5% calf serum (Life Technologies) and six growth factors (thyrotropic hormone, hydrocortisone, insulin, transferrin, somatostatin and glycyl-histidyl-lysine; Sigma, St. Louis, MO). PC Cl3 cells were transfected with control siRNA and siRNA against rat cbx7 as previously reported [11]. Mouse embryonic fibroblasts (MEFs) from cbx7 knockout mice were established, grown and transfected (passage P3 and P4) as described elsewhere [10].

### Protein extraction and western blot analysis

Protein extraction and western blot procedure were carried out as previously reported [11]. After electrophoresis and blotting procedures, nitrocellulose membranes were incubated overnight with the primary antibody in a cold room (+4 °C). Membranes were then incubated with the secondary antibody (horseradish peroxidase-conjugated, 1∶3000) for 60 min (at room temperature) and the reaction was detected with a western blot detection system (GE Healthcare, Little Chalfont, UK). The antibodies used were anti-HA (Roche Applied Science, Mannheim, Germany), anti-CBX7 (sc-70232, Santa Cruz Biotechnology, Inc., Santa Cruz, CA), anti-CBX7 (ab21873, Abcam, Cambridge, UK), anti-His-probe (sc-804, Santa Cruz Biotechnology), anti-Egr1 (sc-189, Santa Cruz Biotechnology, Inc.), anti-c-Fos (sc-52, Santa Cruz Biotechnology, Inc.), anti-Fos B (sc-48, Santa Cruz Biotechnology, Inc.), anti-Tubulin γ (sc-17787, Santa Cruz Biotechnology, Inc.) and anti-β-Actin (Clone AC-15 A5441, Sigma-Aldrich Co., St. Louis, MO).

### Luciferase activity assay

Luciferase transactivation assay was performed as reported elsewhere [11]. A region spanning 1000 bp upstream of the tanscriptional start site (TSS) of the CBX7-regulated genes (primers sequences are available in Dataset S1) was PCR-amplified, and inserted upstream to the open reading frame of the luciferase gene contained in the pGL3 vector (Promega, Fitchburg, WI). All the reporter constructs generated were checked for mutations by direct sequencing. CMV-β-galactosidase expression vector was used to normalize transfection activity according to galactosidase activity. Protein lysates were obtained 36 hours after transfection of HEK 293 cells and luciferase activity was measured for each point by using a Lumat LB9507 luminometer (Berthold Technologies, Bad Wildbad, Germany) and the Dual Light System kit (Life Technologies). All assays were performed in duplicate and results are the mean of three independent experiments.

### Chromatin immunoprecipitation assay (ChIP)

Chromatin samples obtained from cells and tissues were processed for chromatin immunoprecipitation as reported elsewhere [10], [11]. Samples were subjected to immunoprecipitation with anti-CBX7 antibodies (ab21873, Abcam). Non related IgG were used as negative control of the immunoprecipitation. For qPCR 3 µl of 150 µl IP DNA was used and input DNA values were used to normalize the values from quantitative ChIP samples. Percent input was calculated according to the formula 2^ΔCt^×3, where ΔCt is the difference between Ct_input_ and Ct_IP_ [10]. All quantitative data were derived from three independent experiments, and for each experiment qPCR was performed in triplicate. The sequences of the used primers are reported in Dataset S1. The promoter of human and mouse GAPDH gene was used as negative control of the CBX7 chromatin interaction [4].

### Ethics

The ethics committee of the medical faculty and the state medical board of the Centre Hospitalier Lyon Sud, Pierre Bénite, France, agreed to these investigations, and written informed consent was obtained from all of the patients included in this study.

### Statistical analyses

Statistical evaluation were performed by using Graph Pad Prism. Student's t test was used for the comparison between two groups of experiments. Results are expressed as means ± SD and differences were considered to be significant if p<0.05. Distribution of qRT-PCR Fold Change values relative to each gene in papillary thyroid carcinomas and lung carcinomas were obtained and depicted by using the box and whiskers (min to max) and percentile method. For correlation analysis, Fold Change values were used to construct scatter diagrams, to obtain a trend line and to calculate the Pearson r value.

## Results

### Gene expression profile of thyroid carcinoma cells following re-expression of CBX7

**Figure 1:**
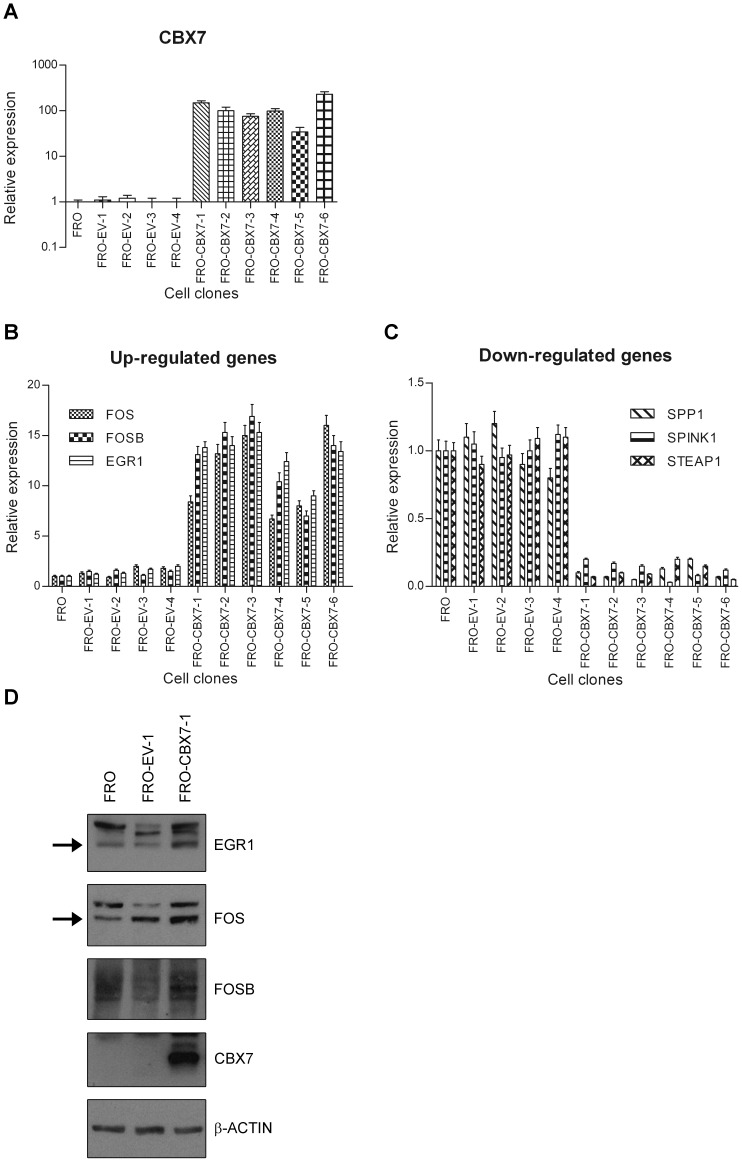
Validation of microarray data. **A**) CBX7 expression was evaluated by qRT-PCR analysis in FRO (wt), and several FRO-EV (empty vector) and FRO-CBX7 cell clones. Values are expressed as Relative expression with respect to the FRO sample that was set equal to 1. **B, C**) The selected CBX7-regulated genes were analyzed by qRT-PCR analysis in FRO (wt), and several FRO-EV and FRO-CBX7 cell clones. FOS, FOSB and EGR1 gene expression was more abundant in FRO-CBX7 cell clones than in the FRO-EV cells, conversely, SPP1, SPINK1 and STEAP1 expression was less pronounced in FRO-CBX7 cells compared with the FRO-EV and FRO (wt) cells. Values are expressed as Relative expression with respect to the FRO sample that was set equal to 1. **D**) The expression of FOS, FOSB and EGR1 was evaluated in one FRO-CBX7 cell clone (FRO-CBX7-1), one FRO-EV (FRO-EV-1) and FRO wild-type cells. β-Actin was evaluated as loading control. Arrows indicate the bands corresponding to FOS and EGR1.

To investigate the mechanism by which the loss of CBX7 expression may contribute to the acquisition of a malignant phenotype, we analysed the gene expression profile of a thyroid carcinoma cell line (FRO) in which the expression of CBX7 was restored (FRO-CBX7, Figure 1A). RNAs extracted from FRO-EV-1 (a clone stably transfected with the empty vector) and FRO-CBX7-1 clone, were hybridized to an Affymetrix oligonucleotide array containing several thousand transcripts. The expression profile of the FRO-CBX7-1 cells was then compared with that of the FRO-EV-1. Several transcripts resulted changed in their expression level between FRO-CBX7-1 and FRO-EV-1 (Table 1 and 2, Table S1 and S2). 53 transcripts (17 up-regulated and 36 down-regulated) had an absolute fold change value more than 2.0 (Table 1 and 2), while 263 transcripts (103 up-regulated and 160 down-regulated) show an absolute fold change value comprised from 1.5 to 2.0 (Table S1 and S2). As a control of microarray analysis, we observed that CBX7 was highly expressed in the FRO-CBX7-1 (Table 1).

**Table 1:** Genes differentially expressed between FRO-CBX7-1 and FRO-EV-1 with a fold change ≥2,0.

Gene Symbol	Fold Change	mRNA Accession	Description
CBX7	58,01	NM_175709	Homo sapiens chromobox homolog 7 (CBX7)
FOS	3,76	NM_005252	Homo sapiens v-fos FBJ murine osteosarcoma viral oncogene homolog (FOS)
LOC93432	2,76	NR_003715	Homo sapiens maltase-glucoamylase-like pseudogene (LOC93432)
FOSB	2,67	NM_006732	Homo sapiens FBJ murine osteosarcoma viral oncogene homolog B (FOSB)
C1orf88	2,66	BC101501	Homo sapiens chromosome 1 open reading frame 88, (cDNA clone MGC:126550)
FLJ25778	2,49	NM_173569	Homo sapiens hypothetical protein FLJ25778 (FLJ25778)
EGR1	2,45	NM_001964	Homo sapiens early growth response 1 (EGR1)
CHN2	2,44	NM_004067	Homo sapiens chimerin (chimaerin) 2 (CHN2)
LOC200383	2,39	BC015442	Homo sapiens similar to Dynein heavy chain at 16F, mRNA (cDNA clone IMAGE:4424085)
NEK5	2,29	NM_199289	Homo sapiens NIMA (never in mitosis gene a)-related kinase 5 (NEK5)
TRIM24	2,16	NM_015905	Homo sapiens tripartite motif-containing 24 (TRIM24)
TPPP3	2,15	NM_016140	Homo sapiens tubulin polymerization-promoting protein family member 3 (TPPP3)
AGR3	2,10	NM_176813	Homo sapiens anterior gradient homolog 3 (Xenopus laevis) (AGR3)
SLC5A1	2,10	NM_000343	Homo sapiens solute carrier family 5 (sodium/glucose cotransporter), member 1 (SLC5A1)
B3GNT5	2,07	NM_032047	Homo sapiens UDP-GlcNAc:betaGal beta-1,3-N-acetylglucosaminyltransferase 5 (B3GNT5)
TXNIP	2,07	NM_006472	Homo sapiens thioredoxin interacting protein (TXNIP)
ACTA2	2,02	NM_001613	Homo sapiens actin, alpha 2, smooth muscle, aorta (ACTA2)

**Table 2:** Genes differentially expressed between FRO-CBX7-1 and FRO-EV-1 with a fold change ≤−2,0.

Gene Symbol	Fold Change	mRNA Accession	Description
SPINK1	−6,91	NM_003122	Homo sapiens serine peptidase inhibitor, Kazal type 1 (SPINK1)
HLA-DMB	−4,87	NM_002118	Homo sapiens major histocompatibility complex, class II, DM beta (HLA-DMB)
SPP1	−4,64	NM_001040058	Homo sapiens secreted phosphoprotein 1 (osteopontin, bone sialoprotein I) (SPP1)
STEAP1	−3,76	NM_012449	Homo sapiens six transmembrane epithelial antigen of the prostate 1 (STEAP1)
SEMA3A	−3,67	NM_006080	Homo sapiens sema domain, short basic domain, secreted, (semaphorin) 3A (SEMA3A)
KLHL5	−3,26	NM_015990	Homo sapiens kelch-like 5 (Drosophila) (KLHL5)
SGPP2	−3,22	NM_152386	Homo sapiens sphingosine-1-phosphate phosphotase 2 (SGPP2)
REG4	−3,10	NM_032044	Homo sapiens regenerating islet-derived family, member 4 (REG4)
NMUR2	−2,94	NM_020167	Homo sapiens neuromedin U receptor 2 (NMUR2)
FGL1	−2,91	NM_201553	Homo sapiens fibrinogen-like 1 (FGL1)
PRKACB	−2,90	NM_182948	Homo sapiens protein kinase, cAMP-dependent, catalytic, beta (PRKACB)
IFITM3	−2,86	NM_021034	Homo sapiens interferon induced transmembrane protein 3 (1-8U) (IFITM3)
FRG1	−2,81	NM_004477	Homo sapiens FSHD region gene 1 (FRG1)
CALD1	−2,73	NM_033138	Homo sapiens caldesmon 1 (CALD1)
CCDC109B	−2,62	NM_017918	Homo sapiens coiled-coil domain containing 109B (CCDC109B)
ANXA1	−2,54	NM_000700	Homo sapiens annexin A1 (ANXA1)
CYP2C18	−2,47	BC096259	Homo sapiens cytochrome P450, family 2, subfamily C, polypeptide 18
TRPS1	−2,47	NM_014112	Homo sapiens trichorhinophalangeal syndrome I (TRPS1)
SYTL5	−2,40	NM_138780	Homo sapiens synaptotagmin-like 5 (SYTL5)
LOC644714	−2,40	BC047037	Homo sapiens, clone IMAGE:5168377
GOLM1	−2,29	NM_016548	Homo sapiens golgi membrane protein 1 (GOLM1)
DSEL	−2,26	NM_032160	Homo sapiens dermatan sulfate epimerase-like (DSEL)
---	−2,25	ENST00000390263	cdna:known chromosome:NCBI36:2:89400496:89400972: −1 gene:ENSG00000211618
AKR1C2	−2,22	NM_205845	Homo sapiens aldo-keto reductase family 1, member C2 (AKR1C2)
IGSF10	−2,22	NM_178822	Homo sapiens immunoglobulin superfamily, member 10 (IGSF10)
---	−2,21	ENST00000396891	cdna:known-ccds chromosome:NCBI36:6:27212987:27222610: −1 gene:ENSG00000197903
---	−2,20	GENSCAN00000055056	cdna:Genscan chromosome:NCBI36:2:91586250:91588295: −1
FN1	−2,19	NM_212482	Homo sapiens fibronectin 1 (FN1)
TNFRSF11B	−2,15	NM_002546	Homo sapiens tumor necrosis factor receptor superfamily, member 11b (TNFRSF11B)
ANTXR2	−2,14	NM_058172	Homo sapiens anthrax toxin receptor 2 (ANTXR2)
GPR110	−2,11	NM_153840	Homo sapiens G protein-coupled receptor 110 (GPR110)
DRAM	−2,10	NM_018370	Homo sapiens damage-regulated autophagy modulator (DRAM)
MID1	−2,10	ENST00000380785	midline 1 (Opitz/BBB syndrome) (MID1)
CADPS2	−2,06	NM_017954	Homo sapiens Ca2+-dependent activator protein for secretion 2 (CADPS2)
PPP2R3A	−2,03	NM_002718	Homo sapiens protein phosphatase 2 (formerly 2A), regulatory subunit B″, alpha (PPP2R3A)
PEG10	−2,03	NM_001040152	Homo sapiens paternally expressed 10 (PEG10)

Among the genes regulated by CBX7 in human thyroid carcinoma cells we concentrated our attention on FOS, FOSB and EGR1 (Table 1) that were positively regulated, and SPP1, SPINK1 and STEAP1 (Table 2) that were, conversely, negatively regulated by CBX7, since these genes are known to play a relevant role in the acquisition of a fully malignant phenotype. In fact, FOS, FOSB and EGR1 are proteins that have been reported to be down-regulated in several human carcinomas suggesting a critical role of these proteins in cancer progression [17]–[19]. On the other hand, SPP1, SPINK1 and STEAP1 are proteins that are up-regulated in several human carcinomas and their overexpression is also associated to cancer progression [20]–[22]. We evaluated the expression of these transcripts by qRT-PCR in several CBX7-expressing FRO clones (Figure 1B and C) compared with FRO-EV clones and with the FRO (wt) cells. For all of them, qRT-PCR analysis confirmed the differential expression associated with the expression of the CBX7 protein (Figure 1B and C). In addition, Western blot experiments confirmed an increased expression of FOS, FOSB and EGR1 proteins in one FRO cell clone stably expressing CBX7 (FRO-CBX7-1) in comparison to FRO-EV (FRO-EV-1) and FRO wt cells (Figure 1D).

### Analysis of the CBX7-regulated genes in rat thyroid cells and MEF expressing or not the cbx7 gene

**Figure 2:**
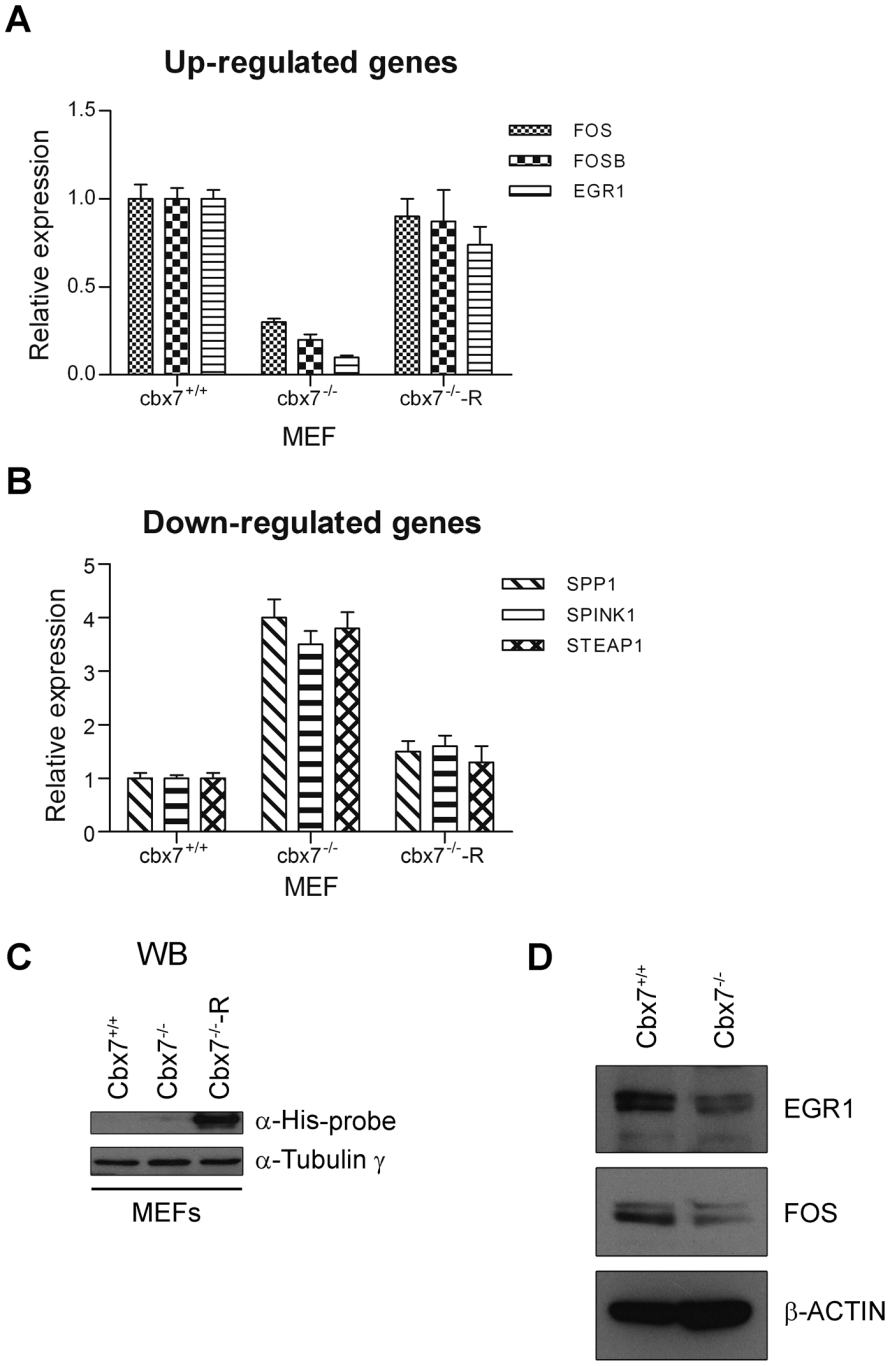
Gene expression in cbx7 knockout MEFs. Quantitative RT-PCR analysis was performed to analyze the expression levels of CBX7-regulated genes in cbx7^+/+^ and cbx7^-/-^ MEFs. Values are expressed as Relative expression with respect to the cbx7^+/+^, that was set equal to 1. **A**) Fos, fosb and egr1 were less expressed in cbx7^-/-^ MEFs compared to the cbx7^+/+^ MEFs. **B**) On the contrary, spp1, spink1 and steap1 genes were more expressed in MEFs cbx7^-/-^ compared to the MEFs cbx7^+/+^. **C**) Cbx7^-/-^ MEFs were transiently transfected with a mammalian vector expressing mouse cbx7 (myc-His-tagged) mRNA (cbx7^-/—^R). Restoration of cbx7 expression is able to revert the phenotype of the cbx7^-/-^ MEFs (**A, B**). Values are expressed as Relative expression with respect to the cbx7^+/+^, that was set equal to 1. **D**) The expression of fos and egr1 proteins was evaluated by western blot in MEFs obtained from cbx7^-/-^ mice in comparison to cbx7^+/+^ MEFs. β-Actin expression was evaluated to normalize protein loading.

We next verified whether the genes differentially expressed in FRO-CBX7 cells showed a differential expression also in MEFs isolated from cbx7 knockout mice [10]. As shown in Figure 2, qRT-PCR analysis confirmed the modulation of the selected CBX7-regulated genes also in this cell system, in fact, genes positively regulated by cbx7 were found down-regulated in cbx7^-/-^ MEFs (Figure 2A), while genes negatively regulated by cbx7, were found up-regulated in the cbx7^-/-^ MEFs, in comparison to cbx7^+/+^ MEFs (Figure 2B). Western blot experiments showed that the expression of fos and egr1 proteins decreases in cbx7^-/-^ MEFs, if compared to cbx7^+/+^ MEFs (Figure 2D).

The restoration of the cbx7 gene expression in cbx7^-/-^ MEFs (Figure 2C) led to an increased expression of fos, fosb and egr1 (Figure 2A) and a decreased expression of spink1, spp1 and steap1 (Figure 2B) confirming the regulation of these genes by CBX7 expression.

**Figure 3:**
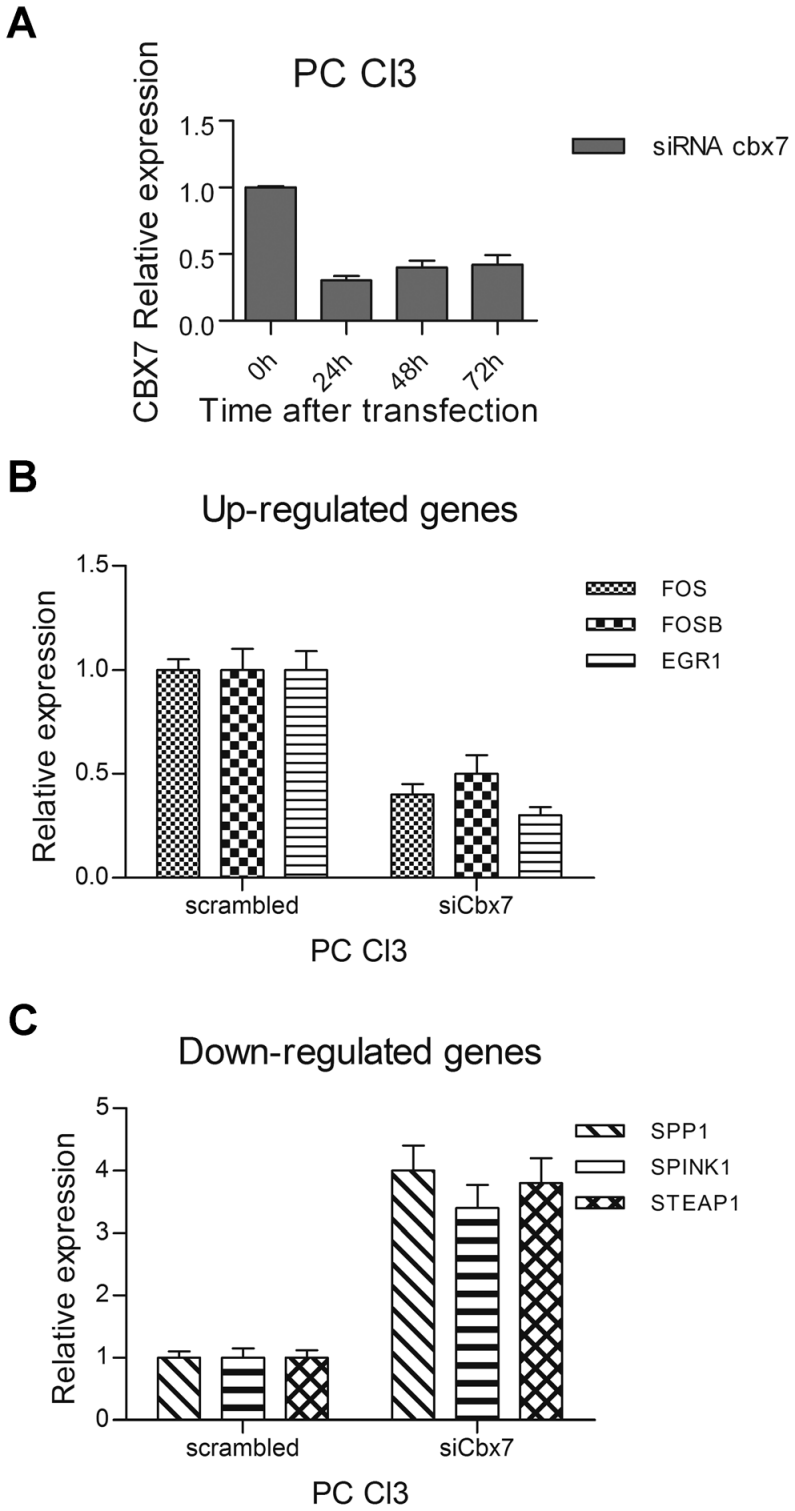
Expression of CBX7-regulated genes in rat normal thyroid cells after suppression of cbx7 expression by RNAi. **A**) PC Cl3 cells were transiently transfected with small interfering RNA (siRNA) against the rat cbx7 mRNA. After transfection we can observe an efficient knockdown of the cbx7 mRNA levels, as evaluated by qRT-PCR analysis. **B, C**) CBX7-regulated genes expression was evaluated by qRT-PCR in rat PC Cl3 cells after transfection with rat cbx7 siRNA. Expression was evaluated 48 hours after transfection. Values are expressed as Relative expression with respect to the PC Cl3 cells transfected with a non silencing control siRNA (scrambled), that were set equal to 1.

To further verify the role of CBX7 in the modulation of these genes, we evaluated their expression in the normal rat thyroid cell line PC Cl3 in which the synthesis of cbx7 was suppressed by RNA interference. The silencing of the cbx7 gene verified at several hours after siRNA treatment (Figure 3A), resulted in the reduction of the CBX7 positively regulated genes (Figure 3B) and in an increased expression of the CBX7 negatively regulated genes (Figure 3C), in comparison with the untreated cells or those treated with the non-silencing control siRNA (scrambled).

### CBX7 binds to promoters of regulated genes and modulates their activity

**Figure 4:**
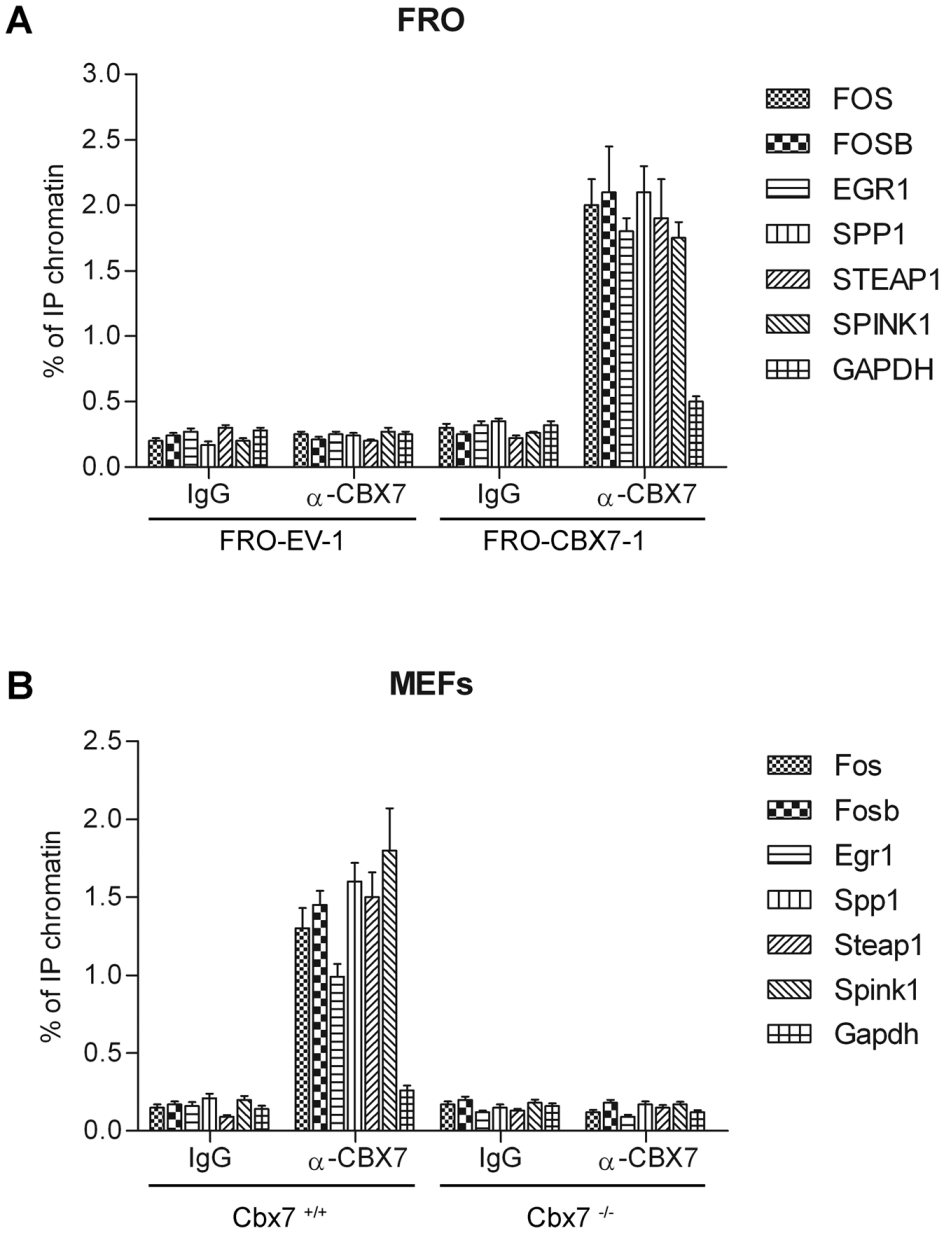
CBX7 binds to the promoters of the CBX7-regulated genes. **A**) FRO-EV-1 and FRO-CBX7-1 cells were subjected to a ChIP assay using antibodies against CBX7. As negative controls, unrelated IgG antibodies were used. The associated DNA was amplified by qPCR using primers specific for the corresponding gene promoter and, as a control of ChIP specificity, primers recognizing the human GAPDH gene promoter. **B**) MEFs obtained from cbx7^+/+^ and cbx7^-/-^ were analyzed for the binding of cbx7 protein to the promoters of its regulated genes. As negative controls, unrelated IgG antibodies were used and, as a control of ChIP specificity, primers recognizing the mouse Gapdh gene promoter were used. Data are reported as percent input and were calculated by using the following formula: 2^ΔCt^×3, where ΔCt is the difference between Ct_input_ and Ct_IP_.

To evaluate whether CBX7 was directly involved in the differential expression of these genes *in vivo*, we performed a chromatin immunoprecipitation (ChIP) assay. Then, FRO-EV-1 and FRO-CBX7-1 cells were crosslinked and immunoprecipitated with anti-CBX7 or IgG antibodies. Immunoprecipitation of chromatin was subsequently analyzed by qPCR using primers spanning the region of these genes promoter (Dataset S1). The results shown in Figure 4A demonstrate that CBX7 was able to bind to these promoters. In fact, anti-CBX7 antibodies precipitated region from the promoter of these genes in FRO-CBX7-1 cells, compared with FRO-EV-1 cells (Figure 4A). Moreover, no enrichment was observed in samples immunoprecipitated with normal rabbit IgG and when primers for the human GAPDH control promoter were used, indicating that the binding is specific for these promoters (Figure 4A). The same result was achieved using chromatin obtained from HEK 293 cells transiently transfected with CBX7 expression vector (Figure S1).

To confirm the binding of endogenous CBX7 protein to the promoters of these genes, we took also advantage of MEFs and tissues obtained from the cbx7 knockout mouse [10]. ChIP experiments were performed on MEFs, kidney and spleen tissues obtained from cbx7^+/+^ and cbx7^-/-^ mice. Anti-CBX7 antibodies were able to recognize endogenous cbx7 and to precipitate chromatin from cbx7^+/+^ but not from cbx7^-/-^ MEFs (Figure 4B) and tissues (Figure S2) confirming the binding of endogenous cbx7 to these promoter regions. These ChIP experiments, therefore, indicated that CBX7 physically interacts with the promoter regions of these genes *in vivo*, therefore directly modulating their expression.

**Figure 5:**
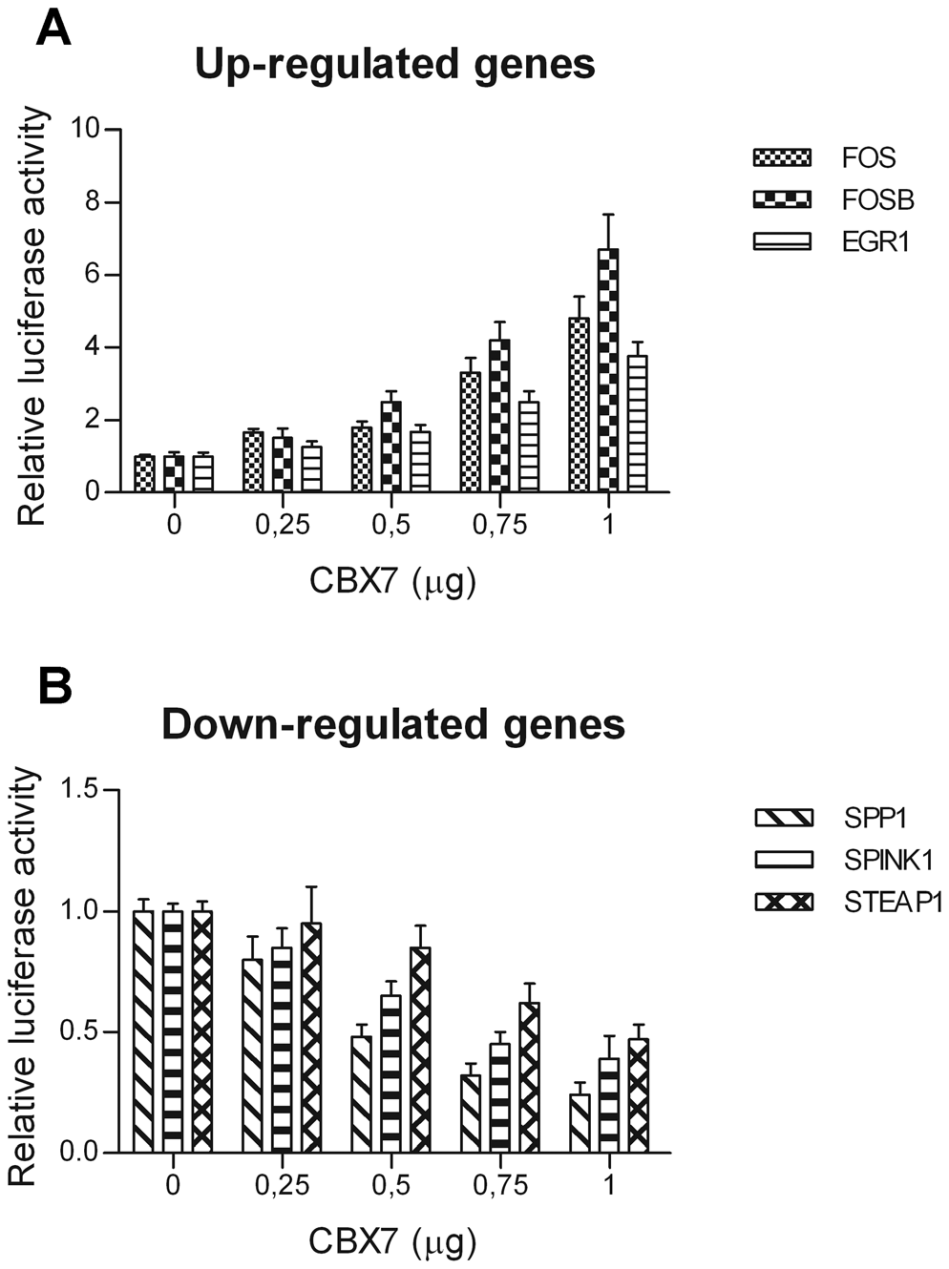
CBX7 modulates the activity of the CBX7-regulate gene promoters. HEK 293 cells were transiently co-transfected with increasing amounts of CBX7 expression vector and a constant amount of vector containing the luciferase gene under the control of the promoters of CBX7-regulated genes. Increasing amounts of CBX7 enforced the expression of the reporter gene under the control of the FOS, FOSB and EGR1 promoter regions (**A**), whereas repressed the activity of the reporter gene under the control of the SPP1, SPINK1 and STEAP1 promoters (**B**).The relative activity of firefly luciferase expression was standardized to a transfection control, using β-galactosidase. The scale bars represent the mean ± SD (n = 3).

Subsequently, to evaluate the effect of CBX7 expression on the transcription of these regulated genes, HEK 293 cells were transiently co-transfected with the expression vector encoding CBX7 and with a reporter vector carrying the luciferase gene under the control of a 1000 bp promoter region located upstream of the TSS of each one of these genes. As shown in Figure 5, CBX7 increased the transcriptional activity of FOS, FOSB and EGR1 promoters (Figure 5A), whereas it repressed the transcriptional activity of SPP1, SPINK1 and STEAP1 promoters (Figure 5B). Moreover, the effects of CBX7 on these promoters was dose dependent. Therefore, ChIP and luciferase assay experiments indicate that CBX7 is able to directly regulate the transcription of the selected CBX7-regulated genes by binding to their promoters.

### The expression of the CBX7-regulated genes correlates with CBX7 expression also in human carcinomas

**Figure 6:**
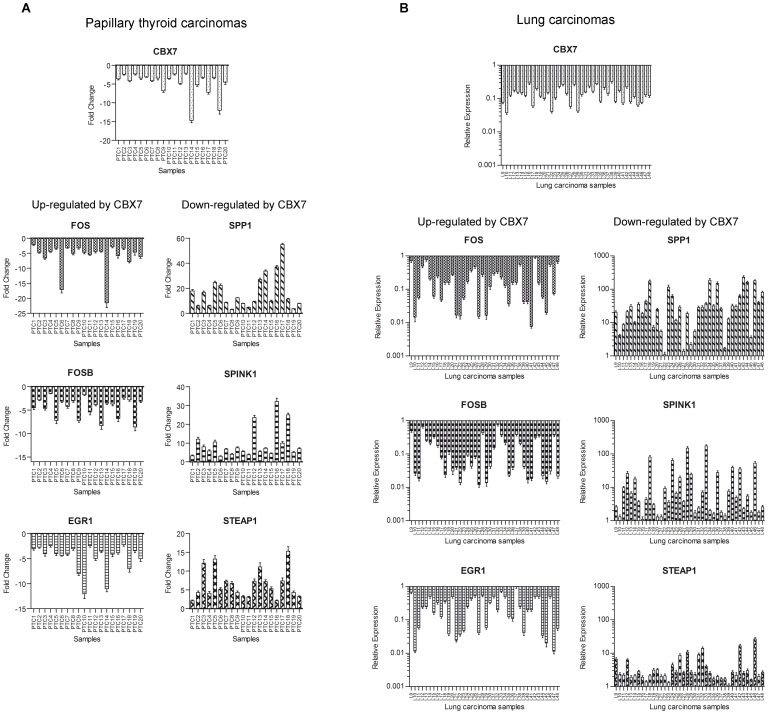
CBX7 and CBX7-regulated gene expression levels are correlated in human carcinoma samples. We analyzed the expression of CBX7, FOS, FOSB, EGR1, SPP1, SPINK1 and STEAP1 by qRT-PCR in papillary thyroid carcinomas (PTC) (**A**) and human lung carcinoma samples (**B**). The expression of FOS, FOSB and EGR1 is down-regulated, as it occurs for CBX7, in the neoplastic tissues with respect to the normal counterparts. Conversely, the expression of SPP1, SPINK1 and STEAP1 was up-regulated in the neoplastic samples with respect to the normal tissues, showing an opposite tendency if compared with that of CBX7. Results are expressed as Fold Change (for PTC) or Relative Expression (for lung carcinomas) with respect to a pool of normal samples which were set equal to 1. The range of variability of CBX7 and CBX7-regulated gene expression in normal thyroid and lung tissues was less than 10%.

Since previous findings showed that CBX7 expression was reduced in thyroid, colon and lung carcinomas [4], [5],[10], with expression levels almost completely undetectable in most advanced thyroid cancers, we hypothesized that the loss of CBX7 gene could be involved in advanced stages of thyroid carcinogenesis by the consequent modulation of these genes. Therefore, we analyzed CBX7 and the selected CBX7-regulated genes in human thyroid and lung carcinomas by qRT-PCR. As far as the papillary thyroid carcinomas are concerned, FOS, FOSB and EGR1 genes were down-regulated, like CBX7, while SPP1, SPINK1 and STEAP1 were up-regulated (Figure 6A). Equally, the expression analysis of these genes in lung carcinomas showed the same behavior (Figure 6B). The correlation analysis in PTC and lung carcinomas (Pearson r) showed the correlation between CBX7 and its regulated genes (PTC: FOS, p = 0.0438; lung carcinomas: FOS, p<0.0001; FOSB, p<0.0001; EGR1, p<0.0021, Figure S3 and S4).

Therefore, these data strongly suggest that the loss of CBX7 gene expression might contribute to the appearance of a malignant phenotype by modulating a set of genes critical for the progression step of carcinogenesis.

## Discussion

It has been previously observed that the reduced CBX7 expression is associated with several human carcinomas, and the complete loss of its expression correlates with the most advanced stages of malignancies [5]–[10]. Therefore, it is reasonable to hypothesize that the loss of expression of CBX7 may play a key role in the cancer progression. Consistently, CBX7 is able to counteract the decreased expression of the E-cadherin gene whose loss of expression represents a feature of the epithelial-mesenchymal transition [11], playing a critical role in maintaining normal epithelial cell morphology [12], [13].

In order to better understand the role of the loss of CBX7 gene expression in cancer progression our work aimed to identify the genes regulated by CBX7. Then, by using an Affymetrix cDNA microarray, we analysed the gene expression profile of thyroid carcinoma cells in which the CBX7 expression was restored, and identified several genes up- and down-regulated. Among the genes up-regulated by CBX7 we concentrated our attention on FOS, FOSB and EGR1.

FOS and FOSB are members of the AP-1 (Activating Protein 1) complex and are able to interact with members of the jun family [23]. FOS has been implicated mainly in signal transduction, cell differentiation and proliferation [24]. Many studies reported that FOS modulated several important genes for tumourigenesis, causing the down-regulation of tumour-suppressor genes [25] and leading to invasive growth of cancer cells [26]. However, some more recent studies suggested that FOS may also have tumour-suppressor activity and might have a function in apoptosis [17], [27].

In addition to FOS, FOSB has also been shown to have a function in progression of various tumour types being down-regulated in poorly differentiated mammary carcinomas [18]. Conversely, FOSL-1 and FOSL-2, whose overexpression leads to enhanced tumour cell motility and invasion in breast cancer, colorectal cancer and mesothelioma [28], were not regulated by CBX7 (data not shown). Therefore, the loss of CBX7 expression could deregulate the composition of the AP-1 complex, which, in turn, could trigger a program of transcriptional alteration that culminate in the appearance of tumours.

EGR1 (early growth response) is a DNA-binding transcription factor strongly and rapidly induced in response to a wide spectrum of stimuli such as serum, growth factors, radiations and stress [29]. Several studies suggest that EGR1 can act as a tumour suppressor gene, being able to suppress the growth of several kind of carcinoma cells [19], [30].

Among the genes that appeared down-regulated by CBX7 expression we focused on the SPP1, SPINK1 and STEAP1 genes since their expression has been found overexpressed in human carcinomas. SPP1 is a secreted, integrin-binding phosphoprotein (osteopontin) which has been associated with tumour progression in multiple tumour types, including breast, hepatocellular, prostate, and colon carcinomas [20], [31], [32]. High osteopontin expression levels resulted associated with breast advanced tumour stage and poor patient prognosis [33]. Experimental studies have shown that the osteopontin contributed functionally to malignancy, by directly influencing cell and tissue properties such as migration and invasion [34], tumour angiogenesis [35], and cell survival through the inhibition of apoptosis [36].

SPINK1 (serine protease inhibitor Kazal type 1) [37] has been reported to modulate cell migration and tissue repair [38]. Recently, it has been found to promote growth in pancreatic cancer by stimulating the epidermal growth factor receptor [21]. Altered SPINK1 expression has been associated with decreased survival in ovarian and colorectal cancer [39], [40]. Moreover, elevated serum and urine concentrations of SPINK1 are associated with adverse prognosis in ovarian [41], kidney [42], colorectal [43] and bladder cancer [44].

STEAP (six-transmembrane epithelial antigen of the prostate) is a membrane protein of 339 aminoacid, characterized by six transmembrane domains [22]. STEAP is highly expressed at all steps of prostate cancer [22] and is also present in numerous human cancer cell lines [45], while its expression is quite low in normal human tissues. The functional involvement of this protein in tumour biology could be duo to its role of transporter protein or ion channel in epithelial cells [46].

The regulation of these genes by CBX7 has been supported by other cell systems. In fact, suppression of cbx7 expression by RNAi experiments in rat normal thyroid cells and analysis of these genes in MEFs deriving from cbx7 knockout mice have confirmed the ability of CBX7 to regulate these genes. Subsequently, chromatin immunoprecipitation studies and evaluation of the activity of the promoters of these genes in presence or absence of CBX7 have shown that CBX7 is able to directly modulate the expression of the selected CBX7-regulated genes by binding to their promoters.

Finally, the analysis of CBX7 and the selected CBX7-regulated genes in human thyroid and lung carcinomas has shown a positive or negative correlation between CBX7 and the selected CBX7-regulated genes suggesting that the loss of CBX7 expression may contribute to the malignant phenotype by the deregulation of genes whose expression is critical for cancer progression.

In conclusion, here we report the identification of a set of genes up- and down-regulated by CBX7, that may contribute to the acquisition of a very aggressive phenotype when their regulation is modified by the loss of the CBX7 expression.

## Supporting Information

Figure S1 **CBX7 binds to the promoters of the CBX7-regulated genes in HEK 293 cells.** **A**) HEK 293 cells transiently transfected with CBX7 expression vector were subjected to a ChIP assay using antibodies against CBX7. As negative controls, unrelated IgG antibodies were used. The associated DNA was amplified by qPCR using primers specific for the corresponding gene promoter and, as a control of ChIP specificity, primers recognizing the human GAPDH gene promoter (Dataset S1). Data are reported as percent input and were calculated by using the following formula: 2^ΔCt^×3, where ΔCt is the difference between Ct_input_ and Ct_IP_. Quantitative PCR was performed in triplicate for each experiment (three independent experiments). **B**) Enforced expression of CBX7 in HEK 293 was evaluated by Western blot analysis using antibodies directed against CBX7.
(DOCX)

Figure S2 **Cbx7 binds to the promoters of the cbx7-regulated genes in mouse spleen and kidney.** Spleen (**A**) and kidney (**B**) tissues obtained from cbx7^+/+^ and cbx7^-/-^ were analyzed for the binding of cbx7 protein to the promoters of its regulated genes. As negative controls, unrelated IgG antibodies were used. Primers used for the amplification of the mouse promoters and mouse Gapdh as control, are reported in Dataset S1. Data are reported as percent input and were calculated by using the following formula: 2^ΔCt^×3, where ΔCt is the difference between Ct_input_ and Ct_IP_. Quantitative PCR was performed in triplicate for each experiment (three independent experiments).
(DOCX)

Figure S3 **Correlation between CBX7 and CBX7-regulated genes in human papillary thyroid carcinomas.** **A**) The expression of CBX7, FOS, FOSB, EGR1, SPP1, SPINK1 and STEAP1 evaluated by qRT-PCR in papillary thyroid carcinomas (PTC) was reported as box and whiskers distribution (min to max). Each box depicted comprises 50% of samples (from 25% percentile to 75% percentile) and its width indicates the distribution of the samples. Whiskers indicate the minimum and the maximum value. The horizontal lane in the box represents the median value of each sample, above or below which, there are 50% of samples. Results are expressed as Fold Change with respect to a pool of normal samples which were set equal to 1. The range of variability of CBX7 and CBX7-regulated genes expression in normal thyroid tissues was less than 10%. **B**) Correlation (Pearson r) was evaluated between the expression of CBX7 and CBX7-regulated genes in PTC. Genes up- and down-regulated by CBX7 are plotted in two separate graphs. For each gene, Fold Change values were plotted in the graph in correspondence to the CBX7 value to generate a scatter diagram. Then, a trend line was extrapolated and the Pearson r value was calculated.
(DOCX)

Figure S4 **Correlation between CBX7 and CBX7-regulated genes in human lung carcinomas.** **A**) The expression of CBX7, FOS, FOSB, EGR1, SPP1, SPINK1 and STEAP1 evaluated by qRT-PCR in lung carcinomas was reported as box and whiskers distribution (min to max). Each box depicted comprises 50% of samples (from 25% percentile to 75% percentile) and its width indicates the distribution of the samples. Whiskers indicate the minimum and the maximum value. The horizontal lane in the box represents the median value of each sample, above or below which, there are 50% of samples. Results are expressed as Fold Change with respect to a pool of normal samples which were set equal to 1. The range of variability of CBX7 and CBX7-regulated gene expression in normal lung tissues was less than 10%. **B**) Correlation (Pearson r) was evaluated between the expression of CBX7 and CBX7-regulated genes in lung carcinomas. Genes up- and down-regulated by CBX7 are plotted in two separate graphs. For each gene, Fold Change values were plotted in the graph in correspondence to the CBX7 value to generate a scatter diagram. Then, a trend line was extrapolated and the Pearson r value was calculated.
(DOCX)

Table S1 **Genes differentially expressed between FRO-CBX7-1 and FRO-EV-1 with a fold change ≥1,5.**
(DOCX)

Table S2 **Genes differentially expressed between FRO-CBX7-1 and FRO-EV-1 with a fold change ≤-1,5.**
(DOCX)

Dataset S1 **Primer sequences.**
(DOC)
