# Supplementary material for: CBX7 Modulates the Expression of Genes Critical for Cancer Progression
Source: PLoS One. 2014 May 27;9(5):e98295. doi: 10.1371/journal.pone.0098295 (PMC4035280; doi:10.1371/journal.pone.0098295)
Supplement: Figure S1 — CBX7 binds to the promoters of the CBX7-regulated genes in HEK 293 cells. A) HEK 293 cells transiently transfected with CBX7 expression vector were subjected to a ChIP assay using antibodies against CBX7. As negative controls, unrelated IgG antibodies were used. The associated DNA was amplified by qPCR using primers specific for the corresponding gene promoter and, as a control of ChIP specificity, primers recognizing the human GAPDH gene promoter (Dataset S1). Data are reported as percent input and were calculated by using the following formula: 2ΔCt×3, where ΔCt is the difference between Ctinput and CtIP. Quantitative PCR was performed in triplicate for each experiment (three independent experiments). B) Enforced expression of CBX7 in HEK 293 was evaluated by Western blot analysis using antibodies directed against CBX7. (DOCX) [file pone.0098295.s001.docx]

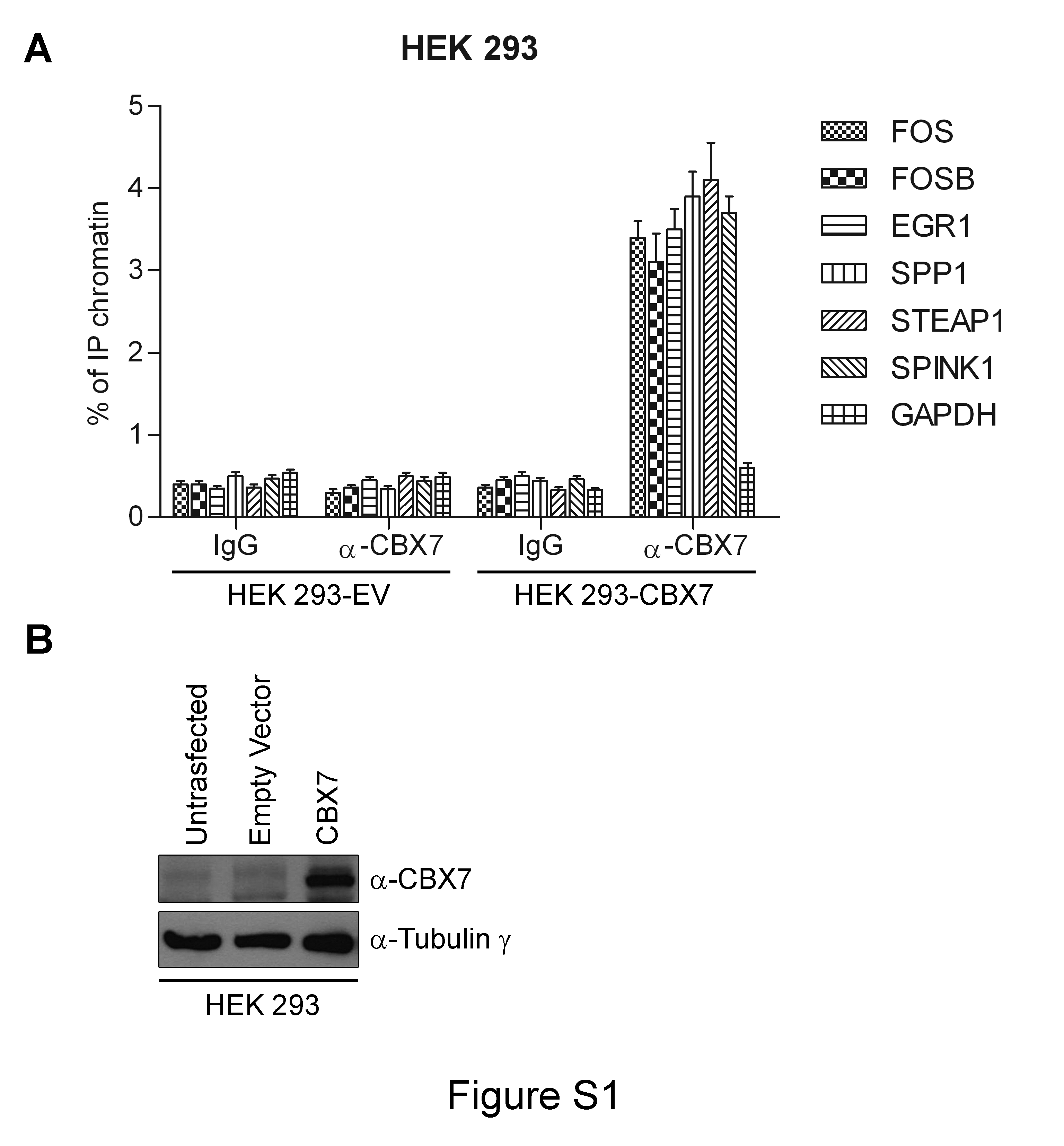


**Figure S1. CBX7 binds to the promoters of the CBX7-regulated genes in HEK 293 cells**

**A)** HEK 293 cells transiently transfected with CBX7 expression vector were subjected to a ChIP assay using antibodies against CBX7. As negative controls, unrelated IgG antibodies were used. The associated DNA was amplified by qPCR using primers specific for the corresponding gene promoter and, as a control of ChIP specificity, primers recognizing the human GAPDH gene promoter (Dataset S1). Data are reported as percent input and were calculated by using the following formula: 2^ΔCt^ × 3, where ΔCt is the difference between Ct_input_ and Ct_IP_. Quantitative PCR was performed in triplicate for each experiment (three independent experiments).

**B)** Enforced expression of CBX7 in HEK 293 was evaluated by Western blot analysis using antibodies directed against CBX7.
