# Supplementary material for: CBX7 Modulates the Expression of Genes Critical for Cancer Progression
Source: PLoS One. 2014 May 27;9(5):e98295. doi: 10.1371/journal.pone.0098295 (PMC4035280; doi:10.1371/journal.pone.0098295)
Supplement: Figure S2 — Cbx7 binds to the promoters of the cbx7-regulated genes in mouse spleen and kidney. Spleen (A) and kidney (B) tissues obtained from cbx7+/+ and cbx7-/- were analyzed for the binding of cbx7 protein to the promoters of its regulated genes. As negative controls, unrelated IgG antibodies were used. Primers used for the amplification of the mouse promoters and mouse Gapdh as control, are reported in Dataset S1. Data are reported as percent input and were calculated by using the following formula: 2ΔCt×3, where ΔCt is the difference between Ctinput and CtIP. Quantitative PCR was performed in triplicate for each experiment (three independent experiments). (DOCX) [file pone.0098295.s002.docx]

**
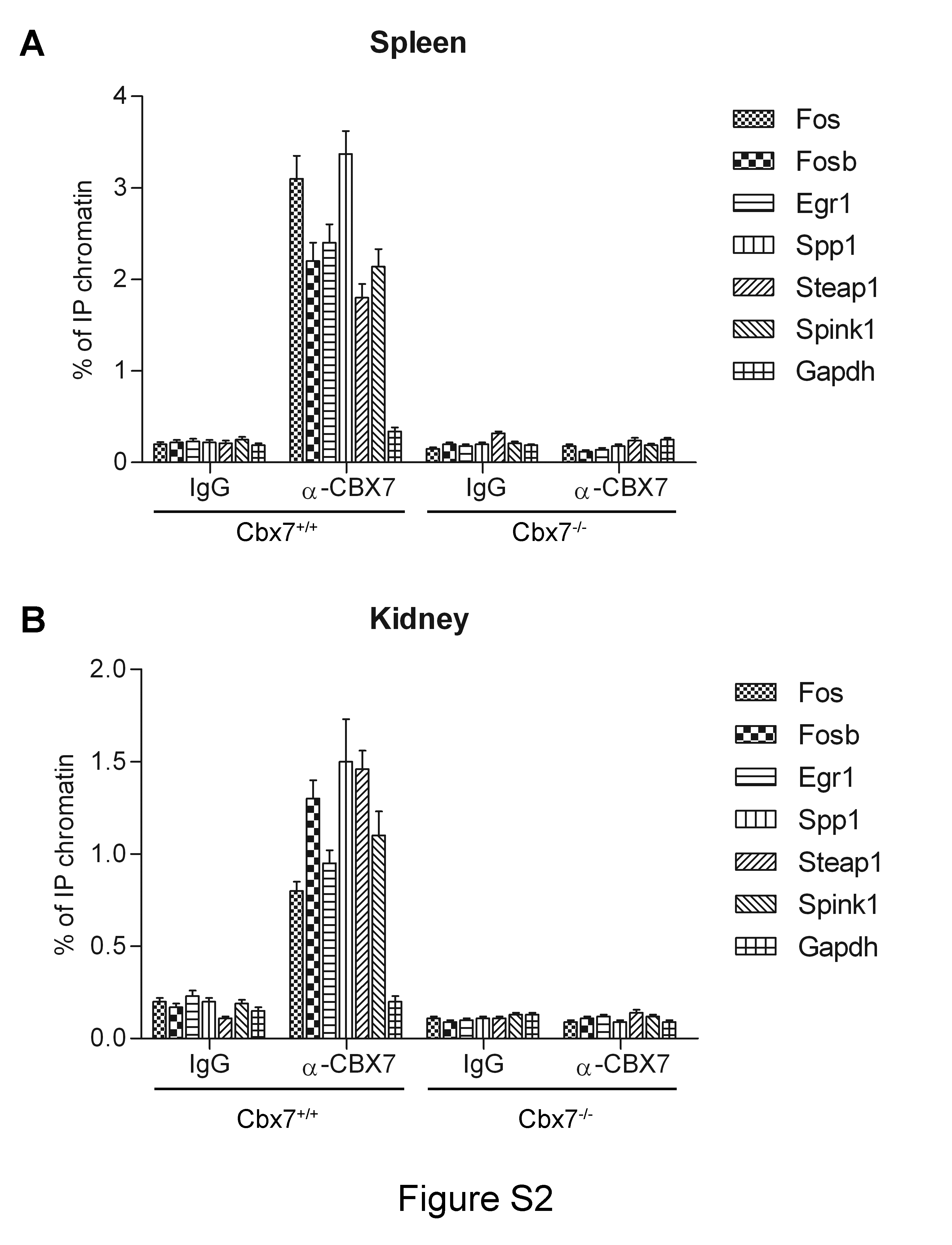
**

**Figure S2.** **Cbx7 binds to the promoters of the cbx7-regulated genes in mouse spleen and kidney**

Spleen **(A)** and kidney **(B)** tissues obtained from cbx7^+/+^ and cbx7^-/-^ were analyzed for the binding of cbx7 protein to the promoters of its regulated genes. As negative controls, unrelated IgG antibodies were used. Primers used for the amplification of the mouse promoters and mouse Gapdh as control, are reported in Dataset S1. Data are reported as percent input and were calculated by using the following formula: 2^ΔCt^ × 3, where ΔCt is the difference between Ct_input_ and Ct_IP_. Quantitative PCR was performed in triplicate for each experiment (three independent experiments).
