# Supplementary material for: CBX7 Modulates the Expression of Genes Critical for Cancer Progression
Source: PLoS One. 2014 May 27;9(5):e98295. doi: 10.1371/journal.pone.0098295 (PMC4035280; doi:10.1371/journal.pone.0098295)
Supplement: Figure S4 — Correlation between CBX7 and CBX7-regulated genes in human lung carcinomas. A) The expression of CBX7, FOS, FOSB, EGR1, SPP1, SPINK1 and STEAP1 evaluated by qRT-PCR in lung carcinomas was reported as box and whiskers distribution (min to max). Each box depicted comprises 50% of samples (from 25% percentile to 75% percentile) and its width indicates the distribution of the samples. Whiskers indicate the minimum and the maximum value. The horizontal lane in the box represents the median value of each sample, above or below which, there are 50% of samples. Results are expressed as Fold Change with respect to a pool of normal samples which were set equal to 1. The range of variability of CBX7 and CBX7-regulated gene expression in normal lung tissues was less than 10%. B) Correlation (Pearson r) was evaluated between the expression of CBX7 and CBX7-regulated genes in lung carcinomas. Genes up- and down-regulated by CBX7 are plotted in two separate graphs. For each gene, Fold Change values were plotted in the graph in correspondence to the CBX7 value to generate a scatter diagram. Then, a trend line was extrapolated and the Pearson r value was calculated. (DOCX) [file pone.0098295.s004.docx]

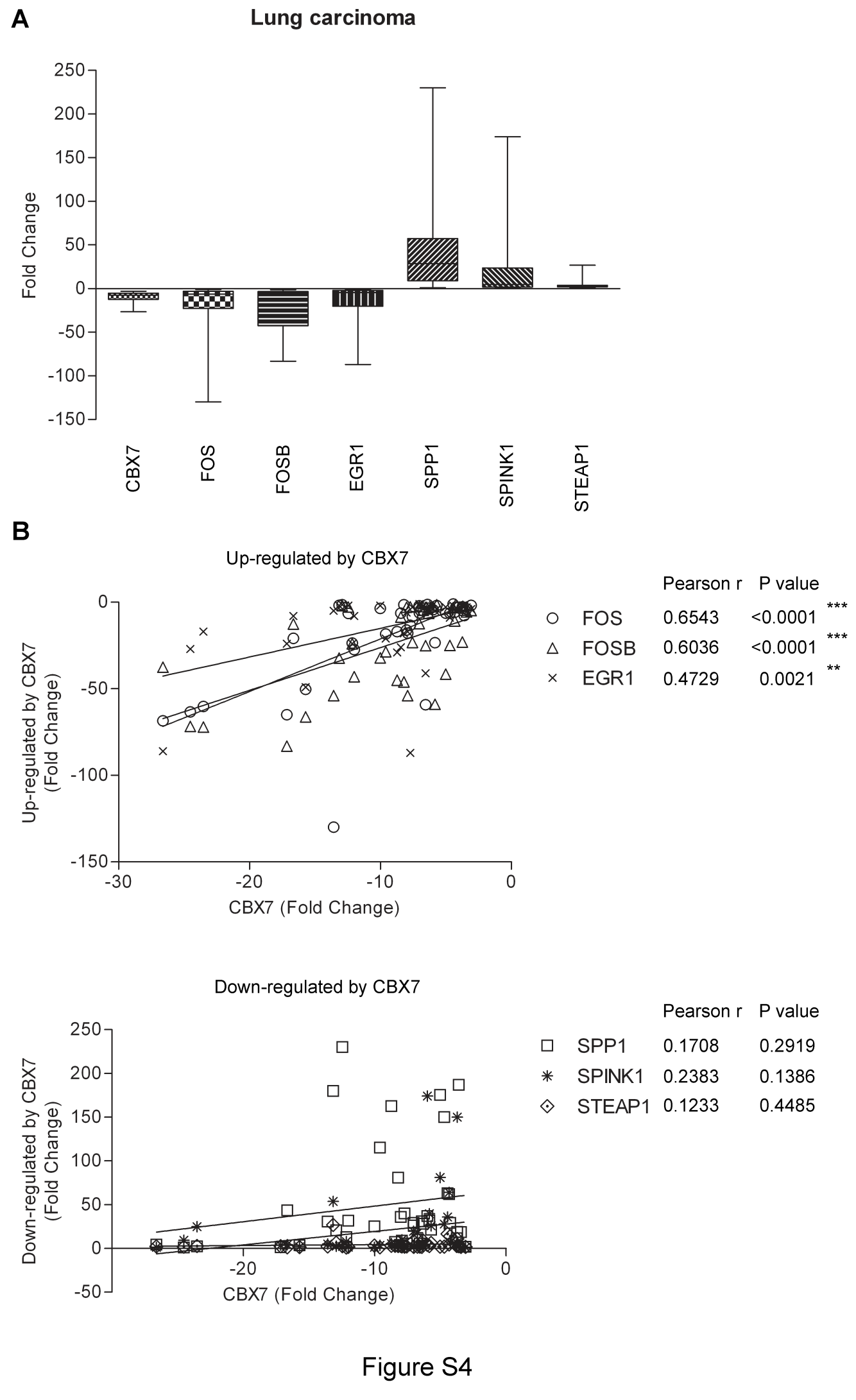


**Figure S4. Correlation between CBX7 and CBX7-regulated genes in human lung carcinomas**

**A)** The expression of CBX7, FOS, FOSB, EGR1, SPP1, SPINK1 and STEAP1 evaluated by qRT-PCR in lung carcinomas was reported as box and whiskers distribution (min to max). Each box depicted comprises 50% of samples (from 25% percentile to 75% percentile) and its width indicates the distribution of the samples. Whiskers indicate the minimum and the maximum value. The horizontal lane in the box represents the median value of each sample, above or below which, there are 50% of samples. Results are expressed as Fold Change with respect to a pool of normal samples which were set equal to 1. The range of variability of CBX7 and CBX7-regulated gene expression in normal lung tissues was less than 10%.

**B)** Correlation (Pearson r) was evaluated between the expression of CBX7 and CBX7-regulated genes in lung carcinomas. Genes up- and down-regulated by CBX7 are plotted in two separate graphs. For each gene, Fold Change values were plotted in the graph in correspondence to the CBX7 value to generate a scatter diagram. Then, a trend line was extrapolated and the Pearson r value was calculated.
