# Supplementary material for: CBX7 Modulates the Expression of Genes Critical for Cancer Progression
Source: PLoS One. 2014 May 27;9(5):e98295. doi: 10.1371/journal.pone.0098295 (PMC4035280; doi:10.1371/journal.pone.0098295)
Supplement: Table S1 — Genes differentially expressed between FRO-CBX7-1 and FRO-EV-1 with a fold change ≥1,5. (DOCX) [file pone.0098295.s005.docx]

| **Table S1.** | |  |  |  |  |  |  |  |  |  |  |
| --- | --- | --- | --- | --- | --- | --- | --- | --- | --- | --- | --- |
| **Genes differentially expressed between FRO-CBX7-1 and FRO-EV-1 with a fold change ≥1,5** | | | | | | |  |  |  |  |  |
|  |  |  |  |  |  |  |  |  |  |  |  |
| **Gene Symbol** | **Fold Change** | **mRNA Accession** | **Description** | |  |  |  |  |  |  |  |
| CBX7 | 58,01 | NM_175709 | Homo sapiens chromobox homolog 7 (CBX7), mRNA. | | | | |  |  |  |  |
| FOS | 3,76 | NM_005252 | Homo sapiens v-fos FBJ murine osteosarcoma viral oncogene homolog (FOS), mRNA. | | | | | | |  |  |
| LOC93432 | 2,76 | NR_003715 | Homo sapiens maltase-glucoamylase-like pseudogene (LOC93432) on chromosome 7. | | | | | | |  |  |
| FOSB | 2,67 | NM_006732 | Homo sapiens FBJ murine osteosarcoma viral oncogene homolog B (FOSB), mRNA. | | | | | | |  |  |
| C1orf88 | 2,66 | BC101501 | Homo sapiens chromosome 1 open reading frame 88, mRNA (cDNA clone MGC:126550 IMAGE:8069007). | | | | | | | | |
| FLJ25778 | 2,49 | NM_173569 | Homo sapiens hypothetical protein FLJ25778 (FLJ25778), mRNA. | | | | | |  |  |  |
| EGR1 | 2,45 | NM_001964 | Homo sapiens early growth response 1 (EGR1), mRNA. | | | | |  |  |  |  |
| CHN2 | 2,44 | NM_004067 | Homo sapiens chimerin (chimaerin) 2 (CHN2), transcript variant 2, mRNA. | | | | | |  |  |  |
| LOC200383 | 2,39 | BC015442 | Homo sapiens similar to Dynein heavy chain at 16F, mRNA (cDNA clone IMAGE:4424085), complete cds. | | | | | | | | |
| NEK5 | 2,29 | NM_199289 | Homo sapiens NIMA (never in mitosis gene a)-related kinase 5 (NEK5), mRNA. | | | | | | |  |  |
| TRIM24 | 2,16 | NM_015905 | Homo sapiens tripartite motif-containing 24 (TRIM24), transcript variant 1, mRNA. | | | | | | |  |  |
| TPPP3 | 2,15 | NM_016140 | Homo sapiens tubulin polymerization-promoting protein family member 3 (TPPP3), mRNA. | | | | | | | |  |
| AGR3 | 2,10 | NM_176813 | Homo sapiens anterior gradient homolog 3 (Xenopus laevis) (AGR3), mRNA. | | | | | | |  |  |
| SLC5A1 | 2,10 | NM_000343 | Homo sapiens solute carrier family 5 (sodium/glucose cotransporter), member 1 (SLC5A1), mRNA. | | | | | | | |  |
| B3GNT5 | 2,07 | NM_032047 | Homo sapiens UDP-GlcNAc:betaGal beta-1,3-N-acetylglucosaminyltransferase 5 (B3GNT5), mRNA. | | | | | | | | |
| TXNIP | 2,07 | NM_006472 | Homo sapiens thioredoxin interacting protein (TXNIP), mRNA. | | | | | |  |  |  |
| ACTA2 | 2,02 | NM_001613 | Homo sapiens actin, alpha 2, smooth muscle, aorta (ACTA2), mRNA. | | | | | |  |  |  |
| LY75 | 1,98 | NM_002349 | Homo sapiens lymphocyte antigen 75 (LY75), mRNA. | | | | |  |  |  |  |
| --- | 1,97 | ENST00000367974 | cdna:known-ccds chromosome:NCBI36:1:159601145:159604288:-1 gene:ENSG00000188931 CCDS30921.1 | | | | | | | | |
| MAK | 1,97 | NM_005906 | Homo sapiens male germ cell-associated kinase (MAK), mRNA. | | | | | |  |  |  |
| BAMBI | 1,94 | NM_012342 | Homo sapiens BMP and activin membrane-bound inhibitor homolog (Xenopus laevis) (BAMBI), mRNA. | | | | | | | | |
| NDRG1 | 1,90 | NM_006096 | Homo sapiens N-myc downstream regulated gene 1 (NDRG1), mRNA. | | | | | |  |  |  |
| FANK1 | 1,89 | AY358154 | Homo sapiens clone DNA176837 CG3104 hlg (UNQ6504) mRNA, complete cds. | | | | | | |  |  |
| KIR2DL3 | 1,88 | NM_014511 | Homo sapiens killer cell immunoglobulin-like receptor, long cytoplasmic tail, 3 (KIR2DL3), transcript variant 1. | | | | | | | | |
| --- | 1,88 | ENST00000385410 | ncrna:Mt_tRNA_pseudogene chromosome:NCBI36:2:202123262:202123310:1 gene:ENSG00000208145 | | | | | | | | |
| CTGF | 1,85 | NM_001901 | Homo sapiens connective tissue growth factor (CTGF), mRNA. | | | | | |  |  |  |
| TTC26 | 1,84 | NM_024926 | Homo sapiens tetratricopeptide repeat domain 26 (TTC26), mRNA. | | | | | |  |  |  |
| JUN | 1,84 | NM_002228 | Homo sapiens jun oncogene (JUN), mRNA. | | | |  |  |  |  |  |
| INSIG1 | 1,83 | NM_198336 | Homo sapiens insulin induced gene 1 (INSIG1), transcript variant 2, mRNA. | | | | | |  |  |  |
| PPIL6 | 1,83 | NM_173672 | Homo sapiens peptidylprolyl isomerase (cyclophilin)-like 6 (PPIL6), mRNA. | | | | | | |  |  |
| CDH3 | 1,81 | NM_001793 | Homo sapiens cadherin 3, type 1, P-cadherin (placental) (CDH3), mRNA. | | | | | |  |  |  |
| LEPREL1 | 1,81 | NM_018192 | Homo sapiens leprecan-like 1 (LEPREL1), mRNA. | | | | |  |  |  |  |
| LOC727963 | 1,78 | XR_015173 | PREDICTED: Homo sapiens similar to elongation factor Tu GTP binding domain containing 1 (LOC727963) | | | | | | | | |
| ZC3HAV1L | 1,78 | NM_080660 | Homo sapiens zinc finger CCCH-type, antiviral 1-like (ZC3HAV1L), mRNA. | | | | | | |  |  |
| EEF1A1 | 1,78 | BC019669 | Homo sapiens eukaryotic translation elongation factor 1 alpha 1, (cDNA clone MGC:25051 IMAGE:4478650). | | | | | | | | |
| CCDC19 | 1,76 | NM_012337 | Homo sapiens coiled-coil domain containing 19 (CCDC19), mRNA. | | | | | |  |  |  |
| MAP3K13 | 1,75 | NM_004721 | Homo sapiens mitogen-activated protein kinase kinase kinase 13 (MAP3K13), mRNA. | | | | | | |  |  |
| IHH | 1,73 | NM_002181 | Homo sapiens Indian hedgehog homolog (Drosophila) (IHH), mRNA. | | | | | |  |  |  |
| WNK4 | 1,73 | NM_032387 | Homo sapiens WNK lysine deficient protein kinase 4 (WNK4), mRNA. | | | | | |  |  |  |
| LUC7L2 | 1,72 | NM_016019 | Homo sapiens LUC7-like 2 (S. cerevisiae) (LUC7L2), mRNA. | | | | |  |  |  |  |
| CLMN | 1,72 | NM_024734 | Homo sapiens calmin (calponin-like, transmembrane) (CLMN), mRNA. | | | | | |  |  |  |
| DNAH7 | 1,71 | NM_018897 | Homo sapiens dynein, axonemal, heavy chain 7 (DNAH7), mRNA. | | | | | |  |  |  |
| SC4MOL | 1,71 | NM_006745 | Homo sapiens sterol-C4-methyl oxidase-like (SC4MOL), transcript variant 1, mRNA. | | | | | | |  |  |
| --- | 1,70 | ENST00000315967 | cdna:known-ccds chromosome:NCBI36:7:138676615:138681605:1 gene:ENSG00000164898 CCDS5853.1 | | | | | | | | |
| COBL | 1,69 | NM_015198 | Homo sapiens cordon-bleu homolog (mouse) (COBL), mRNA. | | | | |  |  |  |  |
| DMXL2 | 1,68 | NM_015263 | Homo sapiens Dmx-like 2 (DMXL2), mRNA. | | | |  |  |  |  |  |
| SEMA6A | 1,68 | NM_020796 | Homo sapiens sema domain, transmembrane domain (TM), and cytoplasmic domain, (semaphorin) 6A (SEMA6A). | | | | | | | | |
| HSD17B7P2 | 1,68 | NR_003086 | Homo sapiens hydroxysteroid (17-beta) dehydrogenase 7 pseudogene 2 (HSD17B7P2) on chromosome 10. | | | | | | | | |
| TCEA3 | 1,68 | NM_003196 | Homo sapiens transcription elongation factor A (SII), 3 (TCEA3), mRNA. | | | | | |  |  |  |
| --- | 1,67 | ENST00000295878 | cdna:known-ccds chromosome:NCBI36:3:115165676:115258150:-1 gene:ENSG00000163617 CCDS2977.1 | | | | | | | | |
| --- | 1,66 | ENST00000355830 | cdna:known chromosome:NCBI36:22:37308300:37376101:-1 gene:ENSG00000184949 | | | | | | | |  |
| TTC25 | 1,65 | NM_031421 | Homo sapiens tetratricopeptide repeat domain 25 (TTC25), mRNA. | | | | | |  |  |  |
| GSTA1 | 1,65 | NM_145740 | Homo sapiens glutathione S-transferase A1 (GSTA1), mRNA. | | | | |  |  |  |  |
| HES1 | 1,64 | NM_005524 | Homo sapiens hairy and enhancer of split 1, (Drosophila) (HES1), mRNA. | | | | | |  |  |  |
| PODXL | 1,64 | NM_001018111 | Homo sapiens podocalyxin-like (PODXL), transcript variant 1, mRNA. | | | | | |  |  |  |
| ENC1 | 1,64 | NM_003633 | Homo sapiens ectodermal-neural cortex (with BTB-like domain) (ENC1), mRNA. | | | | | | |  |  |
| FOXJ1 | 1,63 | NM_001454 | Homo sapiens forkhead box J1 (FOXJ1), mRNA. | | | |  |  |  |  |  |
| HSD17B7 | 1,63 | NM_016371 | Homo sapiens hydroxysteroid (17-beta) dehydrogenase 7 (HSD17B7), mRNA. | | | | | | |  |  |
| NR4A1 | 1,63 | NM_002135 | Homo sapiens nuclear receptor subfamily 4, group A, member 1 (NR4A1), transcript variant 1, mRNA. | | | | | | | | |
| LPIN1 | 1,62 | NM_145693 | Homo sapiens lipin 1 (LPIN1), mRNA. | | | |  |  |  |  |  |
| LYN | 1,62 | NM_002350 | Homo sapiens v-yes-1 Yamaguchi sarcoma viral related oncogene homolog (LYN), mRNA. | | | | | | | |  |
| SPATA17 | 1,62 | NM_138796 | Homo sapiens spermatogenesis associated 17 (SPATA17), mRNA. | | | | | |  |  |  |
| AHNAK2 | 1,62 | NM_138420 | Homo sapiens AHNAK nucleoprotein 2 (AHNAK2), mRNA. | | | | |  |  |  |  |
| --- | 1,61 | BC108718 | Homo sapiens cDNA clone IMAGE:6015397. | | | |  |  |  |  |  |
| TRIM29 | 1,61 | NM_012101 | Homo sapiens tripartite motif-containing 29 (TRIM29), mRNA. | | | | | |  |  |  |
| THBS3 | 1,61 | NM_007112 | Homo sapiens thrombospondin 3 (THBS3), mRNA. | | | | |  |  |  |  |
| RENBP | 1,61 | NM_002910 | Homo sapiens renin binding protein (RENBP), mRNA. | | | | |  |  |  |  |
| HYDIN | 1,61 | NM_032821 | Homo sapiens hydrocephalus inducing homolog (mouse) (HYDIN), transcript variant 1, mRNA. | | | | | | | |  |
| CPNE2 | 1,61 | NM_152727 | Homo sapiens copine II (CPNE2), mRNA. | | | |  |  |  |  |  |
| --- | 1,60 | Z34893 | H.sapiens (RFTS7H) mRNA for immunoglobulin gamma chain variable region, rheumatoid factor (313bp). | | | | | | | | |
| NOX4 | 1,60 | NM_016931 | Homo sapiens NADPH oxidase 4 (NOX4), mRNA. | | | | |  |  |  |  |
| PTK7 | 1,60 | NM_002821 | Homo sapiens PTK7 protein tyrosine kinase 7 (PTK7), transcript variant PTK7-1, mRNA. | | | | | | | |  |
| DPY19L2P2 | 1,60 | NR_003561 | Homo sapiens dpy-19-like 2 pseudogene 2 (C. elegans) (DPY19L2P2) on chromosome 7. | | | | | | | |  |
| USP54 | 1,59 | NM_152586 | Homo sapiens ubiquitin specific peptidase 54 (USP54), mRNA. | | | | | |  |  |  |
| KIAA0828 | 1,59 | NM_015328 | Homo sapiens adenosylhomocysteinase 3 (KIAA0828), mRNA. | | | | | |  |  |  |
| HSPA1B | 1,59 | NM_005346 | Homo sapiens heat shock 70kDa protein 1B (HSPA1B), mRNA. | | | | | |  |  |  |
| OR5M3 | 1,59 | NM_001004742 | Homo sapiens olfactory receptor, family 5, subfamily M, member 3 (OR5M3), mRNA. | | | | | | |  |  |
| MICAL2 | 1,59 | NM_014632 | Homo sapiens microtubule associated monoxygenase, calponin and LIM domain containing 2 (MICAL2). | | | | | | | | |
| PLEKHA2 | 1,58 | NM_021623 | Homo sapiens pleckstrin homology domain containing (phosphoinositide binding specific) member 2 (PLEKHA2). | | | | | | | | |
| LOC200383 | 1,58 | AB051484 | Homo sapiens mRNA for KIAA1697 protein, partial cds. | | | | |  |  |  |  |
| --- | 1,58 | ENST00000328010 | cdna:known chromosome:NCBI36:11:66720294:66720569:-1 gene:ENSG00000184085 | | | | | | | |  |
| KIAA0265 | 1,58 | NM_014997 | Homo sapiens KIAA0265 protein (KIAA0265), mRNA. | | | | |  |  |  |  |
| --- | 1,57 | ENST00000384151 | ncrna:snoRNA chromosome:NCBI36:1:226854806:226854930:1 gene:ENSG00000206878 | | | | | | | |  |
| REPS2 | 1,57 | NM_004726 | Homo sapiens RALBP1 associated Eps domain containing 2 (REPS2), transcript variant 1, mRNA. | | | | | | | |  |
| RSHL3 | 1,57 | NM_001010892 | Homo sapiens radial spokehead-like 3 (RSHL3), mRNA. | | | | |  |  |  |  |
| SLC26A2 | 1,57 | NM_000112 | Homo sapiens solute carrier family 26 (sulfate transporter), member 2 (SLC26A2), mRNA. | | | | | | | |  |
| ZNF716 | 1,55 | XM_938124 | PREDICTED: Homo sapiens zinc finger protein 716 (ZNF716), mRNA. | | | | | |  |  |  |
| SPHK1 | 1,55 | NM_182965 | Homo sapiens sphingosine kinase 1 (SPHK1), transcript variant 2, mRNA. | | | | | |  |  |  |
| ALDOC | 1,55 | NM_005165 | Homo sapiens aldolase C, fructose-bisphosphate (ALDOC), mRNA. | | | | | |  |  |  |
| KIAA0265 | 1,55 | D87454 | Homo sapiens mRNA for KIAA0265 gene, partial cds. | | | | |  |  |  |  |
| DUSP2 | 1,55 | NM_004418 | Homo sapiens dual specificity phosphatase 2 (DUSP2), mRNA. | | | | | |  |  |  |
| DUSP1 | 1,55 | NM_004417 | Homo sapiens dual specificity phosphatase 1 (DUSP1), mRNA. | | | | | |  |  |  |
| SORBS2 | 1,55 | NM_021069 | Homo sapiens sorbin and SH3 domain containing 2 (SORBS2), transcript variant 2, mRNA. | | | | | | | |  |
| UBE2H | 1,55 | NM_003344 | Homo sapiens ubiquitin-conjugating enzyme E2H (UBC8 homolog, yeast) (UBE2H), transcript variant 1, mRNA. | | | | | | | | |
| NR5A2 | 1,54 | NM_205860 | Homo sapiens nuclear receptor subfamily 5, group A, member 2 (NR5A2), transcript variant 1, mRNA. | | | | | | | | |
| MFI2 | 1,54 | NM_005929 | Homo sapiens antigen p97 (melanoma associated) (MFI2), transcript variant 1. | | | | | | |  |  |
| LOC339778 | 1,54 | NM_001105519 | Homo sapiens hypothetical protein LOC339778 (LOC339778), mRNA. | | | | | |  |  |  |
| DNAJB1 | 1,54 | NM_006145 | Homo sapiens DnaJ (Hsp40) homolog, subfamily B, member 1 (DNAJB1), mRNA. | | | | | | |  |  |
| CD207 | 1,54 | NM_015717 | Homo sapiens CD207 molecule, langerin (CD207), mRNA. | | | | |  |  |  |  |
| --- | 1,54 | ENST00000378374 | cdna:known chromosome:NCBI36:11:56036367:56036984:-1 gene:ENSG00000205026 | | | | | | | |  |
| ANXA9 | 1,53 | NM_003568 | Homo sapiens annexin A9 (ANXA9), mRNA. | | | |  |  |  |  |  |
| CRTC3 | 1,53 | NM_022769 | Homo sapiens CREB regulated transcription coactivator 3 (CRTC3), transcript variant 1, mRNA. | | | | | | | |  |
| ACOT4 | 1,53 | NM_152331 | Homo sapiens acyl-CoA thioesterase 4 (ACOT4), mRNA. | | | | |  |  |  |  |
| RARRES1 | 1,53 | NM_206963 | Homo sapiens retinoic acid receptor responder (tazarotene induced) 1 (RARRES1), transcript variant 1, mRNA. | | | | | | | | |
| PLLP | 1,53 | NM_015993 | Homo sapiens plasma membrane proteolipid (plasmolipin) (PLLP), mRNA. | | | | | |  |  |  |
| DTNB | 1,52 | NM_021907 | Homo sapiens dystrobrevin, beta (DTNB), transcript variant 1, mRNA. | | | | | |  |  |  |
| MVK | 1,52 | NM_000431 | Homo sapiens mevalonate kinase (mevalonic aciduria) (MVK), mRNA. | | | | | |  |  |  |
| BTG2 | 1,52 | NM_006763 | Homo sapiens BTG family, member 2 (BTG2), mRNA. | | | | |  |  |  |  |
| LOC221442 | 1,51 | AK126092 | Homo sapiens cDNA FLJ44104 fis, clone TESTI4044123. | | | | |  |  |  |  |
| F5 | 1,51 | NM_000130 | Homo sapiens coagulation factor V (proaccelerin, labile factor) (F5), mRNA. | | | | | | |  |  |
| PIGZ | 1,51 | NM_025163 | Homo sapiens phosphatidylinositol glycan anchor biosynthesis, class Z (PIGZ), mRNA. | | | | | | |  |  |
| RNF208 | 1,51 | NM_031297 | Homo sapiens ring finger protein 208 (RNF208), mRNA. | | | | |  |  |  |  |
| PLA2G10 | 1,51 | NM_003561 | Homo sapiens phospholipase A2, group X (PLA2G10), mRNA. | | | | | |  |  |  |
| TTC7B | 1,51 | NM_001010854 | Homo sapiens tetratricopeptide repeat domain 7B (TTC7B), mRNA. | | | | | |  |  |  |
| C1orf194 | 1,51 | BC127905 | Homo sapiens cDNA clone IMAGE:40132698. | | | |  |  |  |  |  |
| DAPK1 | 1,51 | NM_004938 | Homo sapiens death-associated protein kinase 1 (DAPK1), mRNA. | | | | | |  |  |  |
| HMGCS1 | 1,50 | NM_001098272 | Homo sapiens 3-hydroxy-3-methylglutaryl-Coenzyme A synthase 1 (soluble) (HMGCS1), transcript variant 1. | | | | | | | | |
| DZIP1L | 1,50 | NM_173543 | Homo sapiens DAZ interacting protein 1-like (DZIP1L), mRNA. | | | | | |  |  |  |
| FCHSD2 | 1,50 | NM_014824 | Homo sapiens FCH and double SH3 domains 2 (FCHSD2), mRNA. | | | | | |  |  |  |
| C7orf57 | 1,50 | NM_001100159 | Homo sapiens chromosome 7 open reading frame 57 (C7orf57), mRNA. | | | | | |  |  |  |
