# Supplementary material for: CBX7 Modulates the Expression of Genes Critical for Cancer Progression
Source: PLoS One. 2014 May 27;9(5):e98295. doi: 10.1371/journal.pone.0098295 (PMC4035280; doi:10.1371/journal.pone.0098295)
Supplement: Table S2 — Genes differentially expressed between FRO-CBX7-1 and FRO-EV-1 with a fold change ≤-1,5. (DOCX) [file pone.0098295.s006.docx]

| **Table S2.** | |  |  |  |  |  |  |  |  |
| --- | --- | --- | --- | --- | --- | --- | --- | --- | --- |
| **Genes differentially expressed between FRO-CBX7-1 and FRO-EV-1 with a fold change ≤-1,5** | | | | |  |  |  |  |  |
|  |  |  |  |  |  |  |  |  |  |
| **Gene Symbol** | **Fold Change** | **mRNA Accession** | **Description** |  |  |  |  |  |  |
| SPINK1 | -6,91 | NM_003122 | Homo sapiens serine peptidase inhibitor, Kazal type 1 (SPINK1), mRNA. | | | |  |  |  |
| HLA-DMB | -4,87 | NM_002118 | Homo sapiens major histocompatibility complex, class II, DM beta (HLA-DMB), mRNA. | | | | |  |  |
| SPP1 | -4,64 | NM_001040058 | Homo sapiens secreted phosphoprotein 1 (osteopontin, bone sialoprotein I) (SPP1), transcript variant 1. | | | | | |  |
| STEAP1 | -3,76 | NM_012449 | Homo sapiens six transmembrane epithelial antigen of the prostate 1 (STEAP1), mRNA. | | | | |  |  |
| SEMA3A | -3,67 | NM_006080 | Homo sapiens sema domain, short basic domain, secreted, (semaphorin) 3A (SEMA3A), mRNA. | | | | | |  |
| KLHL5 | -3,26 | NM_015990 | Homo sapiens kelch-like 5 (Drosophila) (KLHL5), transcript variant 1, mRNA. | | | |  |  |  |
| SGPP2 | -3,22 | NM_152386 | Homo sapiens sphingosine-1-phosphate phosphotase 2 (SGPP2), mRNA. | | | |  |  |  |
| REG4 | -3,10 | NM_032044 | Homo sapiens regenerating islet-derived family, member 4 (REG4), mRNA. | | | |  |  |  |
| NMUR2 | -2,94 | NM_020167 | Homo sapiens neuromedin U receptor 2 (NMUR2), mRNA. | | |  |  |  |  |
| FGL1 | -2,91 | NM_201553 | Homo sapiens fibrinogen-like 1 (FGL1), transcript variant 4, mRNA. | | | |  |  |  |
| PRKACB | -2,90 | NM_182948 | Homo sapiens protein kinase, cAMP-dependent, catalytic, beta (PRKACB), transcript variant 1, mRNA. | | | | | |  |
| IFITM3 | -2,86 | NM_021034 | Homo sapiens interferon induced transmembrane protein 3 (1-8U) (IFITM3), mRNA. | | | | |  |  |
| FRG1 | -2,81 | NM_004477 | Homo sapiens FSHD region gene 1 (FRG1), mRNA. | |  |  |  |  |  |
| CALD1 | -2,73 | NM_033138 | Homo sapiens caldesmon 1 (CALD1), transcript variant 1, mRNA. | | |  |  |  |  |
| CCDC109B | -2,62 | NM_017918 | Homo sapiens coiled-coil domain containing 109B (CCDC109B), mRNA. | | | |  |  |  |
| ANXA1 | -2,54 | NM_000700 | Homo sapiens annexin A1 (ANXA1), mRNA. | |  |  |  |  |  |
| CYP2C18 | -2,47 | BC096259 | Homo sapiens cytochrome P450, family 2, subfamily C, polypeptide 18, mRNA, complete cds. | | | | | |  |
| TRPS1 | -2,47 | NM_014112 | Homo sapiens trichorhinophalangeal syndrome I (TRPS1), mRNA. | | |  |  |  |  |
| SYTL5 | -2,40 | NM_138780 | Homo sapiens synaptotagmin-like 5 (SYTL5), mRNA. | | |  |  |  |  |
| LOC644714 | -2,40 | BC047037 | Homo sapiens, clone IMAGE:5168377, mRNA. | |  |  |  |  |  |
| GOLM1 | -2,29 | NM_016548 | Homo sapiens golgi membrane protein 1 (GOLM1), transcript variant 1, mRNA. | | | | |  |  |
| DSEL | -2,26 | NM_032160 | Homo sapiens dermatan sulfate epimerase-like (DSEL), mRNA. | | |  |  |  |  |
| --- | -2,25 | ENST00000390263 | cdna:known chromosome:NCBI36:2:89400496:89400972:-1 gene:ENSG00000211618 | | | | |  |  |
| AKR1C2 | -2,22 | NM_205845 | Homo sapiens aldo-keto reductase family 1, member C2 (AKR1C2), transcript variant 2, mRNA. | | | | | |  |
| IGSF10 | -2,22 | NM_178822 | Homo sapiens immunoglobulin superfamily, member 10 (IGSF10), mRNA. | | | |  |  |  |
| --- | -2,21 | ENST00000396891 | cdna:known-ccds chromosome:NCBI36:6:27212987:27222610:-1 gene:ENSG00000197903 | | | | | |  |
| --- | -2,20 | GENSCAN00000055056 | cdna:Genscan chromosome:NCBI36:2:91586250:91588295:-1 | | |  |  |  |  |
| FN1 | -2,19 | NM_212482 | Homo sapiens fibronectin 1 (FN1), transcript variant 1, mRNA. | | |  |  |  |  |
| TNFRSF11B | -2,15 | NM_002546 | Homo sapiens tumor necrosis factor receptor superfamily, (osteoprotegerin) (TNFRSF11B). | | | | | |  |
| ANTXR2 | -2,14 | NM_058172 | Homo sapiens anthrax toxin receptor 2 (ANTXR2), mRNA. | | |  |  |  |  |
| GPR110 | -2,11 | NM_153840 | Homo sapiens G protein-coupled receptor 110 (GPR110), transcript variant 1, mRNA. | | | | |  |  |
| DRAM | -2,10 | NM_018370 | Homo sapiens damage-regulated autophagy modulator (DRAM), mRNA. | | | |  |  |  |
| MID1 | -2,10 | ENST00000380785 | midline 1 (Opitz/BBB syndrome) (MID1), transcript variant 4, mRNA | | | |  |  |  |
| CADPS2 | -2,06 | NM_017954 | Homo sapiens Ca2+-dependent activator protein for secretion 2 (CADPS2), transcript variant 1, mRNA. | | | | | |  |
| PPP2R3A | -2,03 | NM_002718 | Homo sapiens protein phosphatase 2 (formerly 2A), alpha (PPP2R3A), transcript variant 1. | | | | |  |  |
| PEG10 | -2,03 | NM_001040152 | Homo sapiens paternally expressed 10 (PEG10), transcript variant 1, mRNA. | | | |  |  |  |
| --- | -1,98 | ENST00000296734 | cdna:known-ccds chromosome:NCBI36:5:54491778:54498628:1 gene:ENSG00000164294 | | | | | |  |
| SLC38A4 | -1,98 | NM_018018 | Homo sapiens solute carrier family 38, member 4 (SLC38A4), mRNA. | | | |  |  |  |
| SUCNR1 | -1,96 | NM_033050 | Homo sapiens succinate receptor 1 (SUCNR1), mRNA. | | |  |  |  |  |
| SEMA3C | -1,95 | NM_006379 | Homo sapiens sema domain, immunoglobulin domain (Ig), secreted, (semaphorin) 3C (SEMA3C). | | | | | |  |
| ACAA1 | -1,94 | AK127051 | Homo sapiens cDNA FLJ45108 fis, highly similar to 3-ketoacyl-CoA thiolase. | | | | |  |  |
| C18orf55 | -1,92 | NM_014177 | Homo sapiens chromosome 18 open reading frame 55 (C18orf55), mRNA. | | | |  |  |  |
| --- | -1,92 | ENST00000303735 | cdna:known-ccds chromosome:NCBI36:2:165463058:165520281:-1 gene:ENSG00000169507 | | | | | |  |
| MMP7 | -1,89 | NM_002423 | Homo sapiens matrix metallopeptidase 7 (matrilysin, uterine) (MMP7), mRNA. | | | | |  |  |
| SNAP29 | -1,88 | NM_004782 | Homo sapiens synaptosomal-associated protein, 29kDa (SNAP29), mRNA. | | | |  |  |  |
| RGS2 | -1,87 | NM_002923 | Homo sapiens regulator of G-protein signaling 2, 24kDa (RGS2), mRNA. | | | |  |  |  |
| --- | -1,87 | --- | other_spike |  |  |  |  |  |  |
| QPCT | -1,86 | NM_012413 | Homo sapiens glutaminyl-peptide cyclotransferase (glutaminyl cyclase) (QPCT), mRNA. | | | | |  |  |
| UBE2L3 | -1,86 | ENST00000254302 | ubiquitin-conjugating enzyme E2L 3 (UBE2L3), transcript variant 2, mRNA | | | |  |  |  |
| TBC1D19 | -1,84 | NM_018317 | Homo sapiens TBC1 domain family, member 19 (TBC1D19), mRNA. | | | |  |  |  |
| KYNU | -1,84 | NM_003937 | Homo sapiens kynureninase (L-kynurenine hydrolase) (KYNU), transcript variant 1, mRNA. | | | | |  |  |
| --- | -1,83 | GENSCAN00000000054 | cdna:Genscan chromosome:NCBI36:5:117070532:117160866:-1 | | | |  |  |  |
| SLC2A2 | -1,83 | NM_000340 | Homo sapiens solute carrier family 2 (facilitated glucose transporter), member 2 (SLC2A2), mRNA. | | | | | |  |
| AADAC | -1,81 | NM_001086 | Homo sapiens arylacetamide deacetylase (esterase) (AADAC), mRNA. | | | |  |  |  |
| ZNF223 | -1,80 | NM_013361 | Homo sapiens zinc finger protein 223 (ZNF223), mRNA. | | |  |  |  |  |
| RLN2 | -1,80 | NM_134441 | Homo sapiens relaxin 2 (RLN2), transcript variant 1, mRNA. | | |  |  |  |  |
| KRT23 | -1,79 | NM_015515 | Homo sapiens keratin 23 (histone deacetylase inducible) (KRT23), mRNA. | | | |  |  |  |
| ST3GAL1 | -1,78 | NM_003033 | Homo sapiens ST3 beta-galactoside alpha-2,3-sialyltransferase 1 (ST3GAL1), transcript variant 1. | | | | | |  |
| --- | -1,78 | GENSCAN00000053808 | cdna:Genscan chromosome:NCBI36:1:150638568:150638909:1 | | | |  |  |  |
| DKK1 | -1,78 | NM_012242 | Homo sapiens dickkopf homolog 1 (Xenopus laevis) (DKK1), mRNA. | | | |  |  |  |
| ANKRD29 | -1,78 | NM_173505 | Homo sapiens ankyrin repeat domain 29 (ANKRD29), mRNA. | | |  |  |  |  |
| SSBP2 | -1,77 | NM_012446 | Homo sapiens single-stranded DNA binding protein 2 (SSBP2), mRNA. | | | |  |  |  |
| MRPS18C | -1,76 | NM_016067 | Homo sapiens mitochondrial ribosomal protein S18C (MRPS18C). | | | |  |  |  |
| LOC653156 | -1,76 | XM_001133205 | PREDICTED: Homo sapiens similar to 60S ribosomal protein L21, transcript variant 1 (LOC653156). | | | | | |  |
| TMEM16K | -1,76 | NM_018075 | Homo sapiens transmembrane protein 16K (TMEM16K), mRNA. | | |  |  |  |  |
| --- | -1,75 | GENSCAN00000011541 | cdna:Genscan chromosome:NCBI36:1:202139474:202139824:-1 | | | |  |  |  |
| IL33 | -1,75 | NM_033439 | Homo sapiens interleukin 33 (IL33), mRNA. | |  |  |  |  |  |
| --- | -1,75 | ENST00000363064 | ncrna:snoRNA chromosome:NCBI36:1:85830551:85830627:1 gene:ENSG00000199934 | | | | |  |  |
| SLC16A7 | -1,74 | NM_004731 | Homo sapiens solute carrier family 16, member 7 (monocarboxylic acid transporter 2) (SLC16A7). | | | | | |  |
| C6orf117 | -1,74 | NM_138409 | Homo sapiens chromosome 6 open reading frame 117 (C6orf117), mRNA. | | | |  |  |  |
| SLC7A11 | -1,73 | NM_014331 | Homo sapiens solute carrier family 7, (cationic amino acid transporter, y+ system) (SLC7A11). | | | | | |  |
| DYNLT3 | -1,73 | NM_006520 | Homo sapiens dynein, light chain, Tctex-type 3 (DYNLT3), mRNA. | | | |  |  |  |
| SCIN | -1,72 | BC021090 | Homo sapiens scinderin, mRNA (cDNA clone MGC:31749 IMAGE:4877906), complete cds. | | | | | |  |
| --- | -1,72 | ENST00000296530 | cdna:known-ccds chromosome:NCBI36:4:159265213:159312993:-1 gene:ENSG00000164125 | | | | | |  |
| PECI | -1,72 | NM_206836 | Homo sapiens peroxisomal D3,D2-enoyl-CoA isomerase (PECI), transcript variant 2, mRNA. | | | | | |  |
| --- | -1,71 | ENST00000309047 | cdna:known chromosome:NCBI36:3:130015733:130015936:-1 gene:ENSG00000172797 | | | | | |  |
| DACH1 | -1,69 | NM_080759 | Homo sapiens dachshund homolog 1 (Drosophila) (DACH1), transcript variant 1, mRNA. | | | | |  |  |
| FLRT3 | -1,69 | NM_198391 | Homo sapiens fibronectin leucine rich transmembrane protein 3 (FLRT3), transcript variant 2, mRNA. | | | | | |  |
| TGFB2 | -1,69 | NM_003238 | Homo sapiens transforming growth factor, beta 2 (TGFB2), mRNA. | | | |  |  |  |
| SGCE | -1,69 | NM_001099401 | Homo sapiens sarcoglycan, epsilon (SGCE), transcript variant 1, mRNA. | | | |  |  |  |
| UGT2B7 | -1,68 | BC030974 | Homo sapiens UDP glucuronosyltransferase 2 family, polypeptide B7, (MGC:32620 IMAGE:4593426). | | | | | |  |
| GRAMD3 | -1,68 | NM_023927 | Homo sapiens GRAM domain containing 3 (GRAMD3), mRNA. | | |  |  |  |  |
| LOC643300 | -1,68 | XR_016534 | PREDICTED: Homo sapiens similar to 60 kDa heat shock protein, mitochondrial precursor (Hsp60) | | | | | |  |
| --- | -1,67 | NM_001029945.1 | Homo sapiens chromosome 1 open reading frame 178 (C1orf178), transcript variant c, mRNA | | | | | |  |
| SLC14A1 | -1,67 | NM_015865 | Homo sapiens solute carrier family 14 (urea transporter), member 1 (Kidd blood group) (SLC14A1). | | | | | |  |
| SPINK4 | -1,66 | NM_014471 | Homo sapiens serine peptidase inhibitor, Kazal type 4 (SPINK4), mRNA. | | | |  |  |  |
| SLC19A3 | -1,66 | NM_025243 | Homo sapiens solute carrier family 19, member 3 (SLC19A3), mRNA. | | | |  |  |  |
| UBXD6 | -1,65 | NM_005671 | Homo sapiens UBX domain containing 6 (UBXD6), mRNA. | | |  |  |  |  |
| GPC3 | -1,65 | NM_004484 | Homo sapiens glypican 3 (GPC3), mRNA. | |  |  |  |  |  |
| TDG | -1,64 | NM_003211 | Homo sapiens thymine-DNA glycosylase (TDG), mRNA. | | |  |  |  |  |
| MAOB | -1,64 | NM_000898 | Homo sapiens monoamine oxidase B (MAOB), nuclear gene encoding mitochondrial protein, mRNA. | | | | | |  |
| --- | -1,64 | M37726 | Human mitochondrial Lys-tRNA-aaa. |  |  |  |  |  |  |
| RPL26L1 | -1,64 | NM_016093 | Homo sapiens ribosomal protein L26-like 1 (RPL26L1), mRNA. | | |  |  |  |  |
| PTPN20B/PTPN20A | -1,64 | AY704141 | Homo sapiens non-receptor protein tyrosine phosphatase 20 variant 1 (PTPN20), alternatively spliced. | | | | | |  |
| C11orf74 | -1,64 | DQ121386 | Homo sapiens HEPIS mRNA, complete cds. | |  |  |  |  |  |
| ATP1B3 | -1,63 | NM_001679 | Homo sapiens ATPase, Na+/K+ transporting, beta 3 polypeptide (ATP1B3), mRNA. | | | | |  |  |
| ZNF284 | -1,63 | NM_001037813 | Homo sapiens zinc finger protein 284 (ZNF284), mRNA. | | |  |  |  |  |
| CCDC88A | -1,63 | NM_018084 | Homo sapiens coiled-coil domain containing 88A (CCDC88A), mRNA. | | | |  |  |  |
| PPARGC1A | -1,62 | NM_013261 | Homo sapiens peroxisome proliferator-activated receptor gamma, coactivator 1 alpha (PPARGC1A). | | | | | |  |
| FAM3C | -1,62 | NM_014888 | Homo sapiens family with sequence similarity 3, member C (FAM3C), transcript variant 1, mRNA. | | | | | |  |
| PI4KA | -1,62 | NM_058004 | Homo sapiens phosphatidylinositol 4-kinase, catalytic, alpha (PI4KA), transcript variant 2, mRNA. | | | | | |  |
| CNTNAP3B | -1,62 | BX538190 | Homo sapiens mRNA; cDNA DKFZp686I15204 (from clone DKFZp686I15204). | | | | |  |  |
| ADH1C | -1,62 | NM_000669 | Homo sapiens alcohol dehydrogenase 1C (class I), gamma polypeptide (ADH1C), mRNA. | | | | |  |  |
| LOC400061 | -1,61 | XR_016608 | PREDICTED: Homo sapiens similar to CDK105 protein (LOC400061), mRNA. | | | | |  |  |
| --- | -1,61 | ENST00000339579 | cdna:novel chromosome:NCBI36:16:73203992:73204620:-1 gene:ENSG00000187762 | | | | |  |  |
| TXNDC13 | -1,61 | NM_021156 | Homo sapiens thioredoxin domain containing 13 (TXNDC13), mRNA. | | | |  |  |  |
| --- | -1,61 | ENST00000331888 | cdna:novel chromosome:NCBI36:14:60316449:60316839:1 gene:ENSG00000184902 | | | | |  |  |
| EVI2A | -1,61 | NM_001003927 | Homo sapiens ecotropic viral integration site 2A (EVI2A), transcript variant 1, mRNA. | | | | |  |  |
| --- | -1,60 | ENST00000365465 | ncrna:snoRNA chromosome:NCBI36:12:109418540:109418609:-1 gene:ENSG00000202335 | | | | | |  |
| ELMOD2 | -1,60 | NM_153702 | Homo sapiens ELMO/CED-12 domain containing 2 (ELMOD2), mRNA. | | | |  |  |  |
| SGK1 | -1,60 | NM_005627 | Homo sapiens serum/glucocorticoid regulated kinase (SGK), mRNA. | | | |  |  |  |
| --- | -1,60 | S77921 | tRNA-Thr [human, Parkinson's disease patient PD2, tRNA Mitochondrial Partial Mutant ACA Thr, 66 nt]. | | | | | |  |
| FAM3C | -1,60 | NM_014888 | Homo sapiens family with sequence similarity 3, member C (FAM3C), transcript variant 1, mRNA. | | | | | |  |
| RRP15 | -1,60 | NM_016052 | Homo sapiens ribosomal RNA processing 15 homolog (S. cerevisiae) (RRP15), mRNA. | | | | |  |  |
| LOC441487 | -1,60 | XR_019542 | PREDICTED: Homo sapiens similar to methyltransferase-like protein 1 isoform a (LOC441487). | | | | | |  |
| SLCO2A1 | -1,60 | NM_005630 | Homo sapiens solute carrier organic anion transporter family, member 2A1 (SLCO2A1), mRNA. | | | | | |  |
| ING3 | -1,60 | NM_019071 | Homo sapiens inhibitor of growth family, member 3 (ING3), transcript variant 1, mRNA. | | | | |  |  |
| IL1R2 | -1,59 | NM_004633 | Homo sapiens interleukin 1 receptor, type II (IL1R2), transcript variant 1, mRNA. | | | | |  |  |
| CST1 | -1,59 | NM_001898 | Homo sapiens cystatin SN (CST1), mRNA. | |  |  |  |  |  |
| GCNT3 | -1,59 | NM_004751 | Homo sapiens glucosaminyl (N-acetyl) transferase 3, mucin type (GCNT3), mRNA. | | | | |  |  |
| --- | -1,59 | GENSCAN00000004291 | cdna:Genscan chromosome:NCBI36:19:49130409:49206635:1 | | |  |  |  |  |
| HTR2B | -1,59 | NM_000867 | Homo sapiens 5-hydroxytryptamine (serotonin) receptor 2B (HTR2B), mRNA. | | | |  |  |  |
| PLK2 | -1,59 | NM_006622 | Homo sapiens polo-like kinase 2 (Drosophila) (PLK2), mRNA. | | |  |  |  |  |
| --- | -1,59 | ENST00000387980 | ncrna:Mt_tRNA_pseudogene chromosome:NCBI36:1:55611699:55611765:1 | | | |  |  |  |
| SUMO1P3 | -1,59 | NR_002190 | Homo sapiens SUMO1 pseudogene 3 (SUMO1P3) on chromosome 1. | | | |  |  |  |
| FRG1 | -1,58 | NM_004477 | Homo sapiens FSHD region gene 1 (FRG1), mRNA. | |  |  |  |  |  |
| --- | -1,58 | ENST00000387117 | ncrna:Mt_tRNA_pseudogene chromosome:NCBI36:17:19447252:19447319:1 | | | | |  |  |
| FASTKD3 | -1,58 | NM_024091 | Homo sapiens FAST kinase domains 3 (FASTKD3), mRNA. | | |  |  |  |  |
| ZNF146 | -1,57 | NM_007145 | Homo sapiens zinc finger protein 146 (ZNF146), transcript variant 1, mRNA. | | | |  |  |  |
| PON3 | -1,57 | NM_000940 | Homo sapiens paraoxonase 3 (PON3), mRNA. | |  |  |  |  |  |
| GALNT1 | -1,57 | NM_020474 | Homo sapiens UDP-N-acetyl-alpha-D-galactosamine, (GalNAc-T1) (GALNT1). | | | | |  |  |
| RPP40 | -1,57 | NM_006638 | Homo sapiens ribonuclease P/MRP 40kDa subunit (RPP40), mRNA. | | | |  |  |  |
| CFTR | -1,57 | NM_000492 | Homo sapiens cystic fibrosis transmembrane conductance regulator (CFTR). | | | |  |  |  |
| --- | -1,57 | GENSCAN00000012378 | cdna:Genscan chromosome:NCBI36:X:32133940:32134488:1 | | |  |  |  |  |
| GK | -1,57 | NM_000167 | Homo sapiens glycerol kinase (GK), transcript variant 2, mRNA. | | |  |  |  |  |
| BCL2L15 | -1,57 | NM_001010922 | Homo sapiens BCL2-like 15 (BCL2L15), mRNA. | |  |  |  |  |  |
| SEC11C | -1,57 | NM_033280 | Homo sapiens SEC11 homolog C (S. cerevisiae) (SEC11C), mRNA. | | | |  |  |  |
| TM4SF4 | -1,57 | NM_004617 | Homo sapiens transmembrane 4 L six family member 4 (TM4SF4), mRNA. | | | |  |  |  |
| CCL20 | -1,56 | NM_004591 | Homo sapiens chemokine (C-C motif) ligand 20 (CCL20), mRNA. | | | |  |  |  |
| --- | -1,56 | ENST00000249122 | cdna:known-ccds chromosome:NCBI36:22:19385402:19388889:1 gene:ENSG00000128389 | | | | | |  |
| DOC2B | -1,56 | NM_003585 | Homo sapiens double C2-like domains, beta (DOC2B), mRNA. | | |  |  |  |  |
| UTP15 | -1,56 | NM_032175 | Homo sapiens UTP15, U3 small nucleolar ribonucleoprotein, homolog (S. cerevisiae) (UTP15), mRNA. | | | | | |  |
| APOH | -1,56 | NM_000042 | Homo sapiens apolipoprotein H (beta-2-glycoprotein I) (APOH), mRNA. | | | |  |  |  |
| IFIT5 | -1,56 | NM_012420 | Homo sapiens interferon-induced protein with tetratricopeptide repeats 5 (IFIT5), mRNA. | | | | |  |  |
| tcag7.1260 | -1,56 | ENST00000359579 | similar to aldo-keto reductase family 1, member B10 (LOC441282), mRNA | | | |  |  |  |
| TMEM56 | -1,56 | NM_152487 | Homo sapiens transmembrane protein 56 (TMEM56), mRNA. | | |  |  |  |  |
| COQ3 | -1,56 | NM_017421 | Homo sapiens coenzyme Q3 homolog, methyltransferase (S. cerevisiae) (COQ3), mRNA. | | | | |  |  |
| --- | -1,56 | ENST00000388366 | ncrna:Mt_tRNA_pseudogene chromosome:NCBI36:X:55222147:55222215:1 | | | |  |  |  |
| CDKN3 | -1,55 | NM_005192 | Homo sapiens cyclin-dependent kinase inhibitor 3 (CDKN3). | | |  |  |  |  |
| SGCB | -1,55 | NM_000232 | Homo sapiens sarcoglycan, beta (43kDa dystrophin-associated glycoprotein) (SGCB), mRNA. | | | | | |  |
| GK | -1,55 | NM_203391 | Homo sapiens glycerol kinase (GK), transcript variant 1, mRNA. | | |  |  |  |  |
| TLE4 | -1,55 | NM_007005 | Homo sapiens transducin-like enhancer of split 4 (E(sp1) homolog, Drosophila) (TLE4), mRNA. | | | | | |  |
| --- | -1,55 | ENST00000249122 | cdna:known-ccds chromosome:NCBI36:22:19385402:19388889:1 gene:ENSG00000128389 | | | | | |  |
| --- | -1,55 | NM_001353.5 | Homo sapiens aldo-keto reductase family 1, member C1 (AKR1C1), mRNA | | | |  |  |  |
| TLR4 | -1,55 | NM_138554 | Homo sapiens toll-like receptor 4 (TLR4), mRNA. | |  |  |  |  |  |
| CASP4 | -1,54 | NM_033306 | Homo sapiens caspase 4, apoptosis-related cysteine peptidase (CASP4), transcript variant gamma. | | | | | |  |
| DENND2C | -1,54 | BC063894 | Homo sapiens DENN/MADD domain containing 2C, mRNA (cDNA clone MGC:75543). | | | | |  |  |
| SH3BGRL | -1,54 | NM_003022 | Homo sapiens SH3 domain binding glutamic acid-rich protein like (SH3BGRL), mRNA. | | | | |  |  |
| FRMD6 | -1,54 | NM_001042481 | Homo sapiens FERM domain containing 6 (FRMD6), transcript variant 1, mRNA. | | | | |  |  |
| VEPH1 | -1,54 | NM_024621 | Homo sapiens ventricular zone expressed PH domain homolog 1 (zebrafish) (VEPH1), mRNA. | | | | | |  |
| COMMD8 | -1,54 | NM_017845 | Homo sapiens COMM domain containing 8 (COMMD8), mRNA. | | |  |  |  |  |
| MYBL1 | -1,54 | NM_001080416 | Homo sapiens v-myb myeloblastosis viral oncogene homolog (avian)-like 1 (MYBL1), mRNA. | | | | | |  |
| ZNF224 | -1,54 | NM_013398 | Homo sapiens zinc finger protein 224 (ZNF224), mRNA. | | |  |  |  |  |
| ZNF347 | -1,54 | NM_032584 | Homo sapiens zinc finger protein 347 (ZNF347), mRNA. | | |  |  |  |  |
| UBLCP1 | -1,54 | NM_145049 | Homo sapiens ubiquitin-like domain containing CTD phosphatase 1 (UBLCP1), mRNA. | | | | |  |  |
| hCG_2023776 | -1,54 | XM_939955 | Homo sapiens similar to Heterogeneous nuclear ribonucleoprotein A1 (hnRNP core protein A1) (HDP-1) | | | | | | |
| CRKL | -1,54 | NM_005207 | Homo sapiens v-crk sarcoma virus CT10 oncogene homolog (avian)-like (CRKL), mRNA. | | | | |  |  |
| C9orf102 | -1,54 | NM_020207 | Homo sapiens chromosome 9 open reading frame 102 (C9orf102), transcript variant 1, mRNA. | | | | | |  |
| --- | -1,54 | GENSCAN00000015008 | cdna:Genscan chromosome:NCBI36:4:25233995:25234321:1 | | |  |  |  |  |
| FRRS1 | -1,54 | BC029438 | Homo sapiens cDNA clone IMAGE:4603717, partial cds. | | |  |  |  |  |
| EXOSC8 | -1,53 | NM_181503 | Homo sapiens exosome component 8 (EXOSC8), mRNA. | | |  |  |  |  |
| ZNF234 | -1,53 | NM_006630 | Homo sapiens zinc finger protein 234 (ZNF234), mRNA. | | |  |  |  |  |
| LOC285550 | -1,53 | XR_017908 | PREDICTED: Homo sapiens hypothetical protein LOC285550, transcript variant 1 (LOC285550), misc RNA. | | | | | | |
| ZNF17 | -1,53 | NM_006959 | Homo sapiens zinc finger protein 17 (ZNF17), mRNA. | | |  |  |  |  |
| SERPINE2 | -1,53 | BC042628 | Homo sapiens serpin peptidase inhibitor, clade E (nexin, plasminogen activator inhibitor type 1), member 2 | | | | | | |
| ANAPC10 | -1,53 | NM_014885 | Homo sapiens anaphase promoting complex subunit 10 (ANAPC10), mRNA. | | | |  |  |  |
| ADRA2A | -1,52 | NM_000681 | Homo sapiens adrenergic, alpha-2A-, receptor (ADRA2A), mRNA. | | | |  |  |  |
| --- | -1,52 | ENST00000368656 | cdna:known-ccds chromosome:NCBI36:6:112515367:112530685:1 gene:ENSG00000203778 CCDS34513.1 | | | | | | |
| GIN1 | -1,52 | NM_017676 | Homo sapiens gypsy retrotransposon integrase 1 (GIN1), mRNA. | | |  |  |  |  |
| CCNE2 | -1,52 | NM_057749 | Homo sapiens cyclin E2 (CCNE2), mRNA. | |  |  |  |  |  |
| GGCX | -1,52 | NM_000821 | Homo sapiens gamma-glutamyl carboxylase (GGCX), mRNA. | | |  |  |  |  |
| --- | -1,52 | GENSCAN00000046851 | cdna:Genscan chromosome:NCBI36:12:67322561:67323113:-1 | | |  |  |  |  |
| ALG10B | -1,52 | NM_001013620 | Homo sapiens asparagine-linked glycosylation 10 homolog B (yeast, alpha-1,2-glucosyltransferase) (ALG10B). | | | | | | |
| PROS1 | -1,52 | NM_000313 | Homo sapiens protein S (alpha) (PROS1), mRNA. | |  |  |  |  |  |
| SNX24 | -1,52 | BC069012 | Homo sapiens sorting nexin 24, mRNA (cDNA clone MGC:78539 IMAGE:4585781), complete cds. | | | | | |  |
| LYRM7 | -1,51 | NM_181705 | Homo sapiens Lyrm7 homolog (mouse) (LYRM7), mRNA. | | |  |  |  |  |
| CFL2 | -1,51 | NM_138638 | Homo sapiens cofilin 2 (muscle) (CFL2), transcript variant 2, mRNA. | | | |  |  |  |
| TDGF1 | -1,51 | NM_003212 | Homo sapiens teratocarcinoma-derived growth factor 1 (TDGF1), mRNA. | | | |  |  |  |
| SLC39A8 | -1,51 | NM_022154 | Homo sapiens solute carrier family 39 (zinc transporter), member 8 (SLC39A8), mRNA. | | | | |  |  |
| --- | -1,51 | GENSCAN00000035250 | cdna:Genscan chromosome:NCBI36:5:115581618:115581891:-1 | | | |  |  |  |
| SLITRK6 | -1,51 | NM_032229 | Homo sapiens SLIT and NTRK-like family, member 6 (SLITRK6), mRNA. | | | |  |  |  |
| RPL31P4 | -1,51 | XR_018222 | PREDICTED: Homo sapiens ribosomal protein L31 pseudogene 4 (RPL31P4), mRNA. | | | | |  |  |
| STIM2 | -1,50 | NM_020860 | Homo sapiens stromal interaction molecule 2 (STIM2), mRNA. | | |  |  |  |  |
| --- | -1,50 | ENST00000311489 | cdna:known chromosome:NCBI36:8:82701680:82701739:-1 gene:ENSG00000173078 | | | | |  |  |
| OVOS2 | -1,50 | NM_001080502 | Homo sapiens ovostatin 2 (OVOS2), mRNA. | |  |  |  |  |  |
| FLJ13611 | -1,50 | NR_003545 | Homo sapiens hypothetical protein FLJ13611 (FLJ13611), transcript variant 4, transcribed RNA. | | | | | |  |
