# Supplementary material for: CBX7 Modulates the Expression of Genes Critical for Cancer Progression
Source: PLoS One. 2014 May 27;9(5):e98295. doi: 10.1371/journal.pone.0098295 (PMC4035280; doi:10.1371/journal.pone.0098295)
Supplement: Dataset S1 — Primer sequences. (DOC) [file pone.0098295.s007.doc]

| **Dataset 1. Primer sequences** | | |
| --- | --- | --- |
|  |  |  |
| **Chromatin immunoprecipitation primer sequences** | | |
|  |  |  |
|  |  |  |
| **Human** | prom_HQ_C_FOS_F2 | 5’-TTAGGACATCTGCGTCAGCA-3’ |
|  | prom_HQ_C_FOS_R2 | 5’-GCCTTGGCGCGTGTCCTAATC-3’ |
|  |  |  |
|  | prom_HQ_FOS_B_F1 | 5’-ATGGCTAATTGCGTCACAGG-3’ |
|  | prom_HQ_FOS_B_R1 | 5’-GCACTGTCCAGCAAGAGGTC-3’ |
|  |  |  |
|  | prom_HQ_EGR1_F1 | 5’-CTTATTTGGGCAGCACCTTATTTGG-3’ |
|  | prom_HQ_EGFR1_R1 | 5’-GCTTCGGGGAAGCCTAGA-3’ |
|  |  |  |
|  | prom_H_SPP1_F1 | 5’-AGGCAAGAGTGGTGCAGAT-3’ |
|  | prom_H_SPP1_R1 | 5’-AGCACTTAGGGATCCCATGA-3’ |
|  |  |  |
|  | prom_HQ_SPINK1_F1 | 5’-CCACAACCACAGAGGGAGTT-3’ |
|  | prom_HQ_SPINK1_R1 | 5’-CAGGTTCTGGGAATGTCACC-3’ |
|  |  |  |
|  | prom_HQ_STEAP_F1 | 5’-TAATAAGCCCCCGGGTAATC-3’ |
|  | prom_HQ_STEAP1_R1 | 5’-CCCCTCGCCTTTTGTTTAAT-3’ |
|  |  |  |
|  | prom_HQ_GAPDH_F | 5’-CCCAAAGTCCTCCTGTTTCA-3’ |
|  | prom_HQ_GAPDH_R | 5’-GTCTTGAGGCCTGAGCTACG-3’ |
|  |  |  |
|  |  |  |
| **Mouse** | prom_MQ_C_Fos_F | 5’-CTACACGCGGAAGGTCTAGG-3’ |
|  | prom_MQ_C_Fos_R | 5’-GCGCTCTGTCGTCAACTCTA-3’ |
|  |  |  |
|  | prom_MQ_Fosb_F | 5’-GCCGAGCTCCTTATATGGCTA-3’ |
|  | prom_MQ_Fosb_R | 5’-CACCTGCCCATAGTGTGACC-3’ |
|  |  |  |
|  | prom_MQ_EGR1_F | 5’-CCACTGCTGCTGTTCCAATA-3’ |
|  | prom_MQ_EGR1_R | 5’-AGCCCTCCCATCCAAGAGT-3’ |
|  |  |  |
|  | prom_MQ_SPP1_F | 5’-TCATCCCCATTGATGTTTTTC-3’ |
|  | prom_MQ_SPP1_R | 5’-TGCATTTAGAATCCCGGAAG-3’ |
|  |  |  |
|  | prom_MQ_SPINK1_F | 5’-CTGCCCATCCATTCAGAACT-3’ |
|  | prom_MQ_SPINK1_R | 5’-AGCTGCATTCCTGACATCCT-3’ |
|  |  |  |
|  | prom_MQ_STEAP_F | 5’-GACGGCGTAATCGCTACAGA-3’ |
|  | prom_MQ_STEAP_R | 5’-CGAAATCTGCAGTTGTGCAT-3’ |
|  |  |  |
|  | prom_MQ_GAPDH_F | 5’-TGAGTCCTATCCTGGGAACCATCA-3’ |
|  | prom_MQ_GAPDH_R | 5’-TTTGAAATGTGCACGCACCAAGCG-3’ |
|  |  |  |
|  |  |  |
| **Primer sequences for the cloning of 1 kb promoter region for transactivation assay** | | |
|  |  |  |
|  |  |  |
| **Human** | FOS-1K-L | 5’-ATAACCACACCTCGCACTCC-3’ |
|  | FOS-1K-R | 5’-GGCTCAGTCTTGGCTTCTCA-3’ |
|  |  |  |
|  | FOSB-1K-L | 5’-tggcaagtgccaaatacaag-3’ |
|  | FOSB-1K-R | 5’-TGGCCGTAGCTCTGAGTCTT-3’ |
|  |  |  |
|  | EGR1-1K-L | 5’-GGGACTAGGGAACAGCCTTT-3’ |
|  | EGR1-1K-R | 5’-TGGGATCTCTCGCGACTC-3’ |
|  |  |  |
|  | OPN-1K-L | 5’-TTCCAAAATCGAATCTGTTCC-3’ |
|  | OPN-1K-R | 5’-TGCTGCTGCAGACATCCTC-3’ |
|  |  |  |
|  | SPINK1-1K-L | 5’-ttccacaggccaatttaagg-3’ |
|  | SPINK1-1K-R | 5’-CTGGGACTGGAAGGGTCATA-3’ |
|  |  |  |
|  | STEAP1-1K-L | 5’-GGGAGGGACGGAGTAAACAT-3’ |
|  | STEAP1-1K-R | 5’-TTCAAGGGACTCACCCACTC-3’ |
|  |  |  |
|  |  |  |
| **Primer sequences for qRT-PCR expression analysis** | | |
|  |  |  |
|  |  |  |
| **Human** | FOS-L | 5’-ctaccactcacccgcagact-3’ |
|  | FOS-R | 5’-aggtccgtgcagaagtcct-3’ |
|  |  |  |
|  | FOSB-L | 5’-GGCGGAGGGAGCTGACCGAC-3’ |
|  | FOSB-R | 5’-CGTAGGGGATCTTGCAGCCCG-3’ |
|  |  |  |
|  | EGR1-L | 5’-Agccctacgagcacctgac-3’ |
|  | EGR1-R | 5’-Ggtttggctggggtaactg-3’ |
|  |  |  |
|  | SPP1-L | 5’-GGCCACATGGCTAAACCCTGACC-3’ |
|  | SPP1-R | 5’-TGGAGTCCTGGCTGTCCACAT-3’ |
|  |  |  |
|  | SPINK1-L | 5’-taagtgcggtgcagttttca-3’ |
|  | SPINK1-R | 5’-tgagaagaaagatgcctgttacc-3’ |
|  |  |  |
|  | STEAP1-L | 5’-TGGCAATACTGGCTCTGTTGGCT-3’ |
|  | STEAP1-R | 5’-GCGTGTATTGTGCCCAGTAGAAGGG-3’ |
|  |  |  |
|  | CBX7-L | 5’-Cgagtatctggtgaagtggaaa-3’ |
|  | CBX7-R | 5’-Gggggtccaagatgtgct-3’ |
|  |  |  |
|  | G6PD-L | 5’-ACAGAGTGAGCCCTTCTTCAA-3’ |
|  | G6PD-R | 5’-ATAGGAGTTGCGGGCAAAG-3’ |
|  |  |  |
|  |  |  |
| **Mouse** | qMM6-FOS-L | 5’-CGCAGAGCATCGGCAGAAGGG-3’ |
|  | qMM6-FOS-R | 5’-GATTCCGGCACTTGGCTGCA-3’ |
|  |  |  |
|  | qMM6-FOSB-L | 5’-AGCTCATCACCCTCCGCCGA-3’ |
|  | qMM6-FOSB-R | 5’-CGGGCATTTCCCCGAGACCG-3’ |
|  |  |  |
|  | qMM6-EGR1-L | 5’-GGGAGCCGAGCGAACAACCC-3’ |
|  | qMM6-EGR1-R | 5’-TGATGGGAGGCAACCGAGTCGT-3’ |
|  |  |  |
|  | qMM6-SPP1-L | 5’-GTGGCCCATGAGGCTGCAGT-3’ |
|  | qMM6-SPP1-R | 5’-GCCAGAATCAGTCACTTTCACCGGG-3’ |
|  |  |  |
|  | qMM6-SPINK-L | 5’-TCAGTGCTTTGGCCCTGCTGAGT-3’ |
|  | qMM6-SPINK-R | 5’-TTCTGGGACATCCCGCCACTGC-3’ |
|  |  |  |
|  | qMM6-STEAP1-L | 5’-GCCATCTTGGCTCTCTTGGCTGTG-3’ |
|  | qMM6-STEAP1-R | 5’-CCAAAGCGTGTACTGTGCCCAGA-3’ |
|  |  |  |
|  | QMM6-CBX7-L | 5’-CTGGGAGCCTATGGAGCA-3’ |
|  | QMM6-CBX7-R | 5’-GGCCCATTGGTCAGGTCT-3’ |
|  |  |  |
|  | QMM6-G6PD-L | 5’-CAGCGGCAACTAAACTCAGA-3’ |
|  | QMM6-G6PD-R | 5’-TTCCCTCAGGATCCCACAC-3’ |
|  |  |  |
|  |  |  |
| **Rat** | qRN6-FOS-L | 5’-CAGCATGGGCTCCCCTGTCAAC-3’ |
|  | qRN6-FOS-R | 5’-CACTGCAGGTCTGGGCTGGTG-3’ |
|  |  |  |
|  | qRN-FOSB-L | 5’-AACTTTGACACCTCGTCCCGGGGC-3’ |
|  | qRN-FOSB-R | 5’-TCCTCTTCGGGAGACAGCTGCTGG-3’ |
|  |  |  |
|  | qRN6-EGR1-L | 5’-CCTCAAGGGGAGCCGAGCGAA-3’ |
|  | qRN6-EGR1-R | 5’-GGAGGCAACCGGGTAGTTTGGC-3’ |
|  |  |  |
|  | qRN-SPP1-L | 5’-GCTTTGCAGTCTCCTGCGGCAA-3’ |
|  | qRN-SPP1-R | 5’-AGACAGGAGGCAAGGCCGAAC-3’ |
|  |  |  |
|  | qRN6-SPINK-L | 5’-TCAGTGCTTTGGCCCTGCTCAATT-3’ |
|  | qRN6-SPINK-R | 5’-TCCCTGGGGCATCCAATAAGTGTA-3’ |
|  |  |  |
|  | qRN6-STEAP1-L | 5’-AGCCTAAGGGGAACCTGGAAGATGA-3’ |
|  | qRN6-STEAP1-R | 5’-TGGACCGTGTGCGGCAAAGG-3’ |
|  |  |  |
|  | qRN6-CBX7-L | 5’-ATCCGGAAGAAGCGCGTGCG-3’ |
|  | qRN6-CBX7-R | 5’-GGCCATGACAAGGCGAGGGTC-3’ |
|  |  |  |
|  | qRN6-G6PD-L | 5’-GTGGCCATGGAAAAGCCTGCCT-3’ |
|  | qRN6-G6PD-R | 5’-TGGGGTTCCCCACATACTGGCC-3’ |
|  |  |  |
|  |  |  |
